# Vasodilator-stimulated phosphoprotein promotes liver metastasis of gastrointestinal cancer by activating a β1-integrin-FAK-YAP1/TAZ signaling pathway

**DOI:** 10.1038/s41698-017-0045-7

**Published:** 2018-01-23

**Authors:** Xiaoyu Xiang, Yuanguo Wang, Hongbin Zhang, Jinhua Piao, Selvaraj Muthusamy, Lei Wang, Yibin Deng, Wei Zhang, Rui Kuang, Daniel D. Billadeau, Shengbing Huang, Jinping Lai, Raul Urrutia, Ningling Kang

**Affiliations:** 1 Tumor Microenvironment and Metastasis, The Hormel Institute, University of Minnesota, Austin, MN 55912 USA; 2 Department of Pathology, St. Louis University, School of Medicine, St. Louis, MO 63104 USA; 3 Cell Death and Cancer Genetics, The Hormel Institute, University of Minnesota, Austin, MN 55912 USA; 4 Department of Computer Science and Engineering, University of Minnesota, Minneapolis, MN 55455 USA; 5 Oncology Research, Mayo Clinic, Rochester, MN 55905 USA; 6 GI Research Unit, Mayo Clinic, Rochester, MN 55905 USA

## Abstract

Extracellular matrix (ECM)-induced β1-integrin-FAK signaling promotes cell attachment, survival, and migration of cancer cells in a distant organ so as to enable cancer metastasis. However, mechanisms governing activation of the β1-integrin-FAK signaling remain incompletely understood. Here, we report that vasodilator-stimulated phosphoprotein (VASP), an actin binding protein, is required for ECM–mediated β1-integrin-FAK-YAP1/TAZ signaling in gastrointestinal (GI) cancer cells and their liver metastasis. In patient-derived samples, VASP is upregulated in 53 of 63 colorectal cancers and 43 of 53 pancreatic ductal adenocarcinomas and high VASP levels correlate with liver metastasis and reduced patient survival. In a Matrigel-based 3-dimensional (3D) culture model, short hairpin RNA (shRNA)–mediated VASP knockdown in colorectal cancer cells (KM12L4, HCT116, and HT29) and pancreatic cancer cells (L3.6 and MIA PaCa-1) suppresses the growth of 3D cancer spheroids. Mechanistic studies reveal that VASP knockdown suppresses FAK phosphorylation and YAP1/TAZ protein levels, but not Akt or Erk-related pathways and that YAP1/TAZ proteins are enhanced by the β1-integrin-FAK signaling. Additionally, VASP regulates the β1-integrin-FAK-YAP1/TAZ signaling by at least two mechanisms: (1) promoting ECM-mediated β1-integrin activation and (2) regulating YAP1/TAZ dephosphorylation at downstream of RhoA to enhance the stability of YAP1/TAZ proteins. In agreement with these, preclinical studies with two experimental liver metastasis mouse models demonstrate that VASP knockdown suppresses GI cancer liver metastasis, β1-integrin activation, and YAP1/TAZ levels of metastatic cancer cells. Together, our data support VASP as a treatment target for liver metastasis of colorectal and pancreatic cancers.

## Liver metastasis: Protein that drives tumor spread offers therapeutic target

A protein involved in cytoskeleton regulation and cell motility control offers a new drug target for cancer spreading to the liver. Ningling Kang Ph.D. from the Hormel Institute in Austin, Minnesota, USA, and colleagues showed that levels of this actin-binding protein, known as vasodilator-stimulated phosphoprotein (VASP), are elevated in most patients with advanced colon and pancreatic cancers and that higher VASP expression levels are linked to liver metastasis and poorer patients’ outcomes. To explore the reasons why, the researchers studied three-dimensional tumor spheroids and mouse metastasis models of these cancers, and identified the signaling pathway by which VASP promotes the survival of cancer cells in distant organs, such as the liver. What’s more, they showed that knocking down VASP of cancer cells in metastasis mouse models suppressed cancer metastatic growth in the liver, suggesting that the same might be true in patients as well.

## Introduction

Integrins are transmembrane receptors for extracellular matrix (ECM) components such as fibronectin, vitronectin, and collagen. They are composed of 24 heterodimers resulting from combinations of each of 18 α subunits and 8 β subunits.^1^ Integrins sense substrate mechanical cues and convert them into biochemical signals involving Erk, Jun *N*-terminal kinase (JNK), and Rho-family small GTPases.^2^ In patients, increased expression of β1-integrin-coupled signaling effectors is associated with the initiation and progression of cancer.^3,4^ In mice, ablation of β1-integrin in mammary gland epithelium impairs tumorigenesis, confirming a requirement of β1-integrin-mediated signaling for tumorigenesis.^5^ Since cell adhesion to ECM is a critical determinant of cancer metastasis,^6^ the β1-integrin-FAK signaling has been proposed as a prometastatic pathway enabling cancer cells to colonize a distant organ.^7–9^ However, how the β1-integrin-FAK signaling is activated and how it regulates metastasis of cancer cells disseminated into the liver, remain incompletely understood.

VASP belongs to Ena/VASP family of proteins that control actin polymerization, cell adhesions, and migration of cells. It has an Ena/VASP homology 1 (EVH1), an Ena/VASP homology 2 (EVH2) and a central proline-rich region domain (PRR) that facilitate specific protein–protein interactions and actin binding characteristics to promote an anti-capping/branching effect on actin filaments.^10,11^ Through blocks A and B of EVH2 domain, VASP binds to G-actin and F-actin respectively to promote F-actin assembly.^12^ The F-actin assembly activity of VASP, however, is influenced by VASP phosphorylation by protein kinase A (PKA), protein kinase G (PKG), and AMP-activated protein kinase (AMPK).^13–15^ Thus, VASP connects and transmits intracellular signaling cascades to cytoskeleton of the cell.

Studies using genetically engineered mouse models demonstrate that VASP promotes cell–cell adhesion of endothelial cells and inhibits adhesion and aggregation of platelets.^16,17^ In vitro experiments reveal that VASP promotes cancer cell migration and proliferation.^18^ However, it is unknown if VASP regulates the β1-integrin-FAK signaling and GI cancer liver metastasis. YAP1/TAZ are transcriptional coactivators that promote TEAD/TEF dependent gene transcription^19–21^ and their functions are regulated by RhoA or F-actin.^20,22,23^ These observations led us to test if YAP1/TAZ represent as a missing link between the β1-integrin-FAK signaling and cancer metastasis and if the functions of YAP1/TAZ are regulated by VASP.

To test these hypotheses, we first performed VASP immunohistochemistry (IHC) and Kaplan–Meier survival analysis for patient samples and found that VASP was upregulated in the majority of colorectal cancers (CRCs) and pancreatic ductal adenocarcinomas (PDACs) and that high VASP levels correlated with liver metastasis and reduced patient survival. Using a Matrigel-based 3D culture model and two experimental liver metastasis mouse models, we demonstrated that VASP was required for the ECM–mediated β1-integrin-FAK-YAP1/TAZ signaling and GI cancer liver metastasis in vitro and in mice. Furthermore, VASP regulated the β1-integrin-FAK-YAP1/TAZ signaling by at least two mechanisms: (1) promoting β1-integrin activation and (2) inducing YAP1/TAZ dephosphorylation to enhance their protein stability in cells. Thus, VASP of cancer cells represents as a therapeutic target for metastatic CRCs and PDACs.

## Results

### VASP is upregulated in the majority of CRCs and PDACs and high VASP levels correlate with liver metastasis and reduced patient survival

**Fig. 1:**
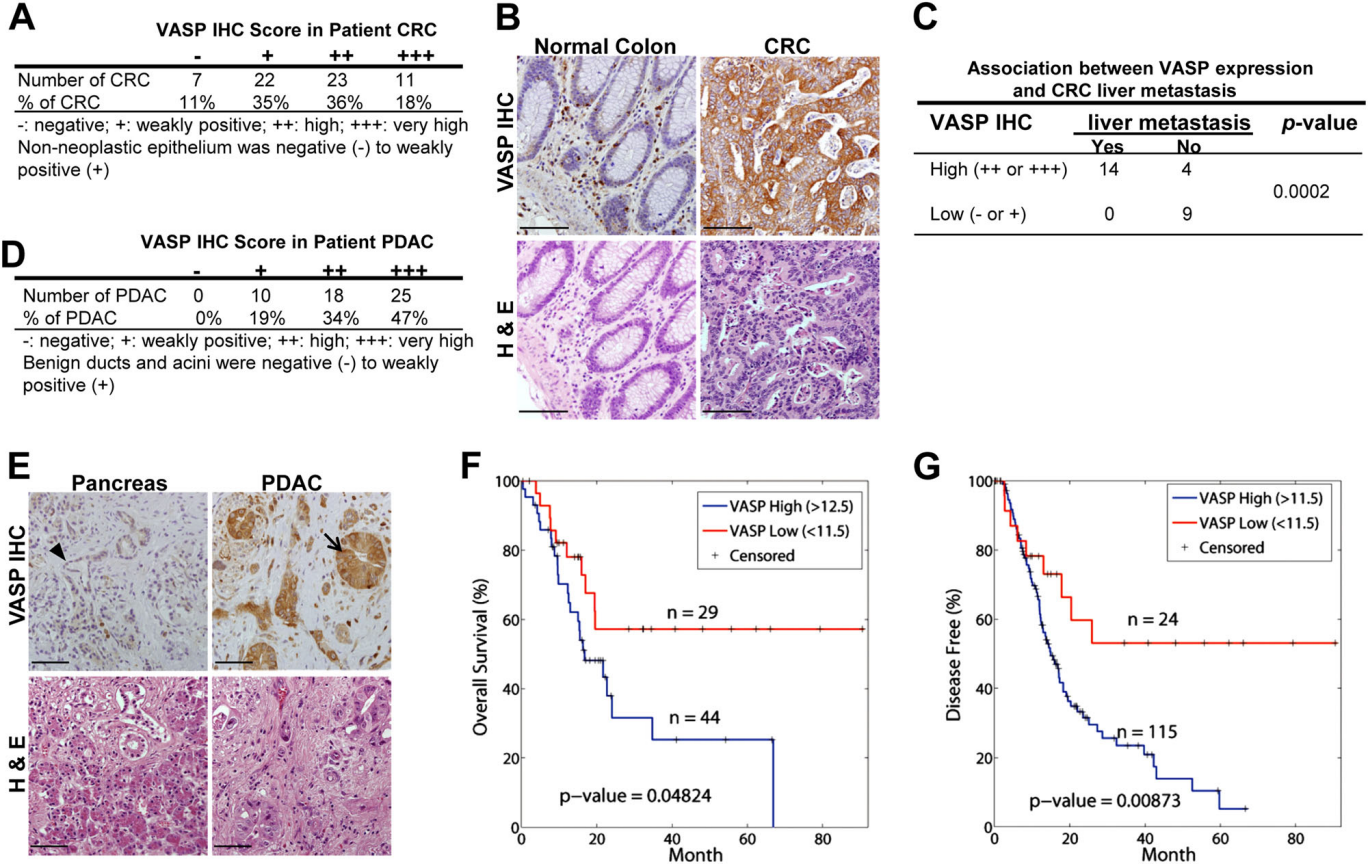
High VASP levels correlate with liver metastasis and reduced patient survival. **a** VASP IHC scores were signed for 63 CRCs of patients. **b** Representative IHC and H & E images of a CRC show that VASP immunoreactivity markedly enhanced in CRC cells as compared to the benign colonic tissues. Bar, 100 µm. **c** VASP IHC data of 27 patients with paired primary CRCs and liver biopsies are shown. In CRCs with high levels of VASP, 14 out of 18 cases (78.8%) had metastases in the liver. In CRCs with low levels of VASP, 0 out of 9 cases (0%) had metastases in the liver. *P* = 0.0002 by Fisher’s exact test. **d** Distribution of VASP IHC scores of 53 PDACs of patients is shown. **e** Representative VASP IHC and H & E images of a PDAC show that VASP immunoreactivity markedly increased in PDAC cells (arrow) as compared to epithelial cells of a benign duct (arrowhead). Bar, 100 µm. **f**, **g** Kaplan–Meier survival analysis revealed that high expression levels of VASP correlated with reduced overall and disease-free survival of PDAC patients. *P* < 0.05 by Log-rank test, *n* is shown in the graphs

To understand if VASP levels correlate with liver metastasis and patient survival, we first performed VASP IHC for CRC tissues of 63 patients. This cohort of patients consisted of 34 females and 29 males with ages ranging from 33 to 100 years. They were diagnosed as colorectal adenocarcinoma and subsequently underwent colorectal resection. VASP immunoreactivity was negative in 7 CRC cases (−, 11%), weakly positive in 22 cases (+, 35%), high in 23 cases (++, 36%), and very high in 11 cases (+++, 18%) (Fig. 1a). Non-neoplastic colorectal epithelia were negative or weakly positive for VASP (− to +). As compared to matched benign tissues, VASP protein was upregulated in 53 CRCs (84%) in this cohort. Presentative VASP IHC data of a patient are shown in Fig. 1b. We also analyzed IHC data of 27 patients with paired primary CRCs and liver biopsies and found that in CRCs expressing high levels of VASP (++ to +++), 14 out of 18 cases (77.8%) had CRC metastases in the liver (Fig. 1c). In contrast, CRCs expressing low levels of VASP (− to +), 0 out of 9 cases (0%) had CRC metastases in the liver (*p* < 0.05 by Fisher exact test, *n* = 27). Thus, VASP is upregulated in the majority of CRCs with its levels correlated with CRC liver metastasis.

We next performed VASP IHC for a cohort of 53 PDACs. This cohort of patients consisted of 22 females and 31 males with ages ranging from 43 to 82 years. They were diagnosed as pancreatic ductal adenocarcinoma and underwent partial pancreatic resection. VASP immunoreactivity was weakly positive in 10 cases (+, 19%), high in 18 cases (++, 34%), and very high in 25 cases (+++, 47%) (Fig. 1d). Benign ducts and acini were negative to weakly positive for VASP (− to +). As compared to matched benign ducts, VASP protein was upregulated in 43 PDACs (81%) in this cohort. Representative VASP IHC data of a patient are shown in Fig. 1e, showing that VASP immunoreactivity was negative in benign pancreatic ductal epithelial cells (arrowhead) but strongly positive in PDAC cancer cells (arrow). Due to aggressiveness and lethality nature of PDACs, we did not obtain enough cases to analyze the correlation between VASP and PDAC liver metastasis. However, we did observe that in PDACs expressing high levels of VASP (++ to +++), 24 out of 26 cases (92%) displayed lymphovascular invasion and 30 of 34 cases (88%) displayed perineural invasion, indicating that VASP promotes metastasis of PDACs. Next, RNA sequencing data of 150 PDACs and their clinical information were downloaded from the Cancer Genome Atlas (TCGA) through the Xena Public Data Hubs (https://xena.ucsc.edu). Kaplan–Meier survival curves were generated for two groups of patients: (1) patients with low VASP gene expressing PDACs (<11.5) and (2) patients with high VASP gene expressing PDACs (>11.5 or >12.5).^24^ When we compared patient survival, patients with low VASP expressing PDACs had significantly higher overall survival and disease-free survival as compared to those with high VASP expressing PDACs (*p* < 0.05 by Log-rank test) (Fig. 1f, g). Together, our clinical data support that high VASP levels of GI cancer cells correlate with cancer metastasis and reduced patient survival.

### ShRNA-based VASP knockdown reduces the growth of cancer spheroids on Matrigel ECM

**Fig. 2:**
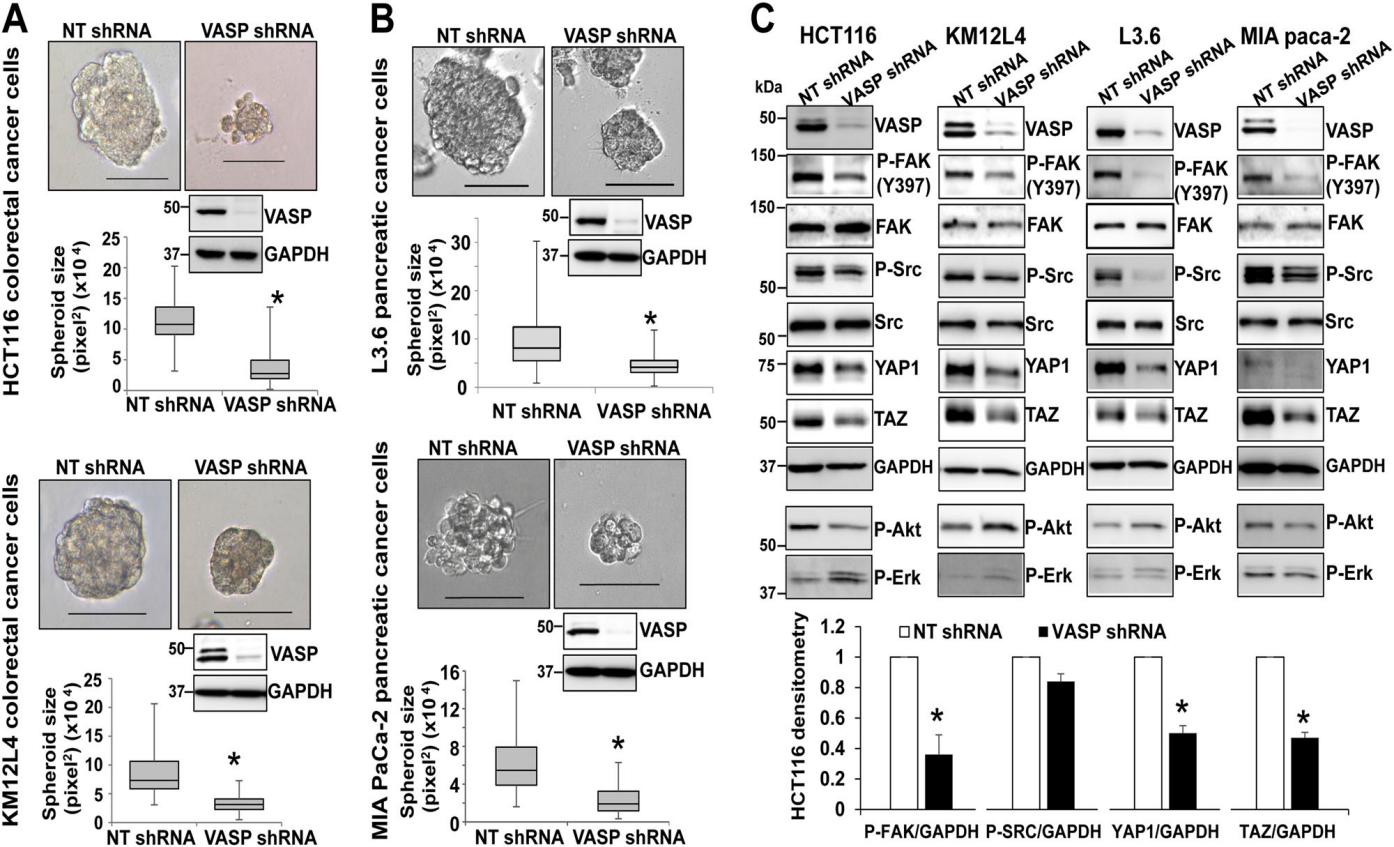
VASP knockdown suppresses the growth of 3D cancer spheroids by reducing P-FAK and YAP1/TAZ protein levels. **a** VASP knockdown in two human CRC cell lines significantly reduced the size of cancer spheroids. *p < 0.05 by *t*-test, *n* > 50 per group; Bar, 100 µm. **b** VASP knockdown in two PDAC cancer cell lines significantly reduced the size of cancer spheroids. *p < 0.05 by *t*-test, *n* > 50 per group; Bar, 100 µm. **c** Cancer spheroids were harvested for WB. VASP knockdown in 4 cell lines consistently reduced phosphorylation of FAK and Src and protein levels of YAP1/TAZ. The effect of VASP knockdown on phosphorylation of Akt or Erk was not consistent and it did not influence the total FAK and Src protein levels. Quantitative data of HCT116 are shown on the bottom. *p < 0.05, by *t*-test, *n* = 3. Samples derived from the same experiment and gels/blots were processed in parallel. Error bar: S.D

Since high VASP levels correlate with liver metastasis and reduced patient survival, we attempted to find novel mechanisms by which VASP promotes liver metastasis of GI cancer cells. Although VASP plays a role in cell proliferation and migration through regulating actin dynamics, this study focused on the role of VASP in ECM-mediated adhesion and survival signals because the initial ECM-mediated signals in cancer cells disseminated into the liver are a key for liver metastasis development. To this end, we plated control cancer cells and VASP knockdown cells on growth factor-reduced Matrigel matrix to induce 3D cancer spheriods^7^ and used this 3D culture model to analyze the influence of VASP on cancer/ECM interactions and ECM-induced pro-survival signals. Multiple cell lines, including CRC cell lines (KM12L4, HCT116, and HT29) and PDAC cell lines (L3.6 and MIA PaCa-1), were used in 3D culture studies aiming to identify a common mechanism underlying liver metastasis. For each cell line, cells were transduced with lentiviruses encoding either NT shRNA (control) or VASP shRNA, made as a single cell suspension, and plated onto Matrigel matrix. The formation and size of 3D cancer spheroids were analyzed 1 week later. As shown in Fig. 2a, b and Suppl. Figure 1a, VASP knockdown in KM12L4, HCT116, HT29, L3.6, and MIA PaCa-1 cells consistently reduced the size of cancer spheroids as compared to control cells (*P* < 0.05 by *t*-test, *n* > 50 spheroids per group). Two different VASP shRNAs (NM_003370.3-1294s1c1 and NM_003370.3-1805s1c1; Sigma-Aldrich, St. Louis, MO) were used and both generated consistent results. Thus, VASP is required for the growth of 3D cancer spheroids on ECM.

### Inactivation of VASP suppresses β1-integrin-FAK signaling and YAP1/TAZ protein levels in 3D culture model

The data of 3D culture led us to determine pro-survival signaling pathway(s) affected by shRNA-based inactivation of VASP. To this end, cancer spheroids were collected for Western blot analyses (WB) to compare phosphorylation levels of Akt, Erk, FAK, and Src. Interestingly, VASP knockdown did not reliably influence the phosphorylation status of Akt or Erk (Fig. 2c). In contrast, it reduced the levels of FAK phosphorylation and YAP1/TAZ in all four cell lines tested but with no influence on the total FAK and Src protein levels (Fig. 2c). Data of HCT116 WB were summarized and shown in Fig. 2c as well (*p* < 0.05 by *t*-test, *n* = 3 repeats). Thus, VASP preferentially influences ECM-activated β1-integrin-FAK signaling but not Akt or Erk-related pathways in 3D culture model.

**Fig. 3:**
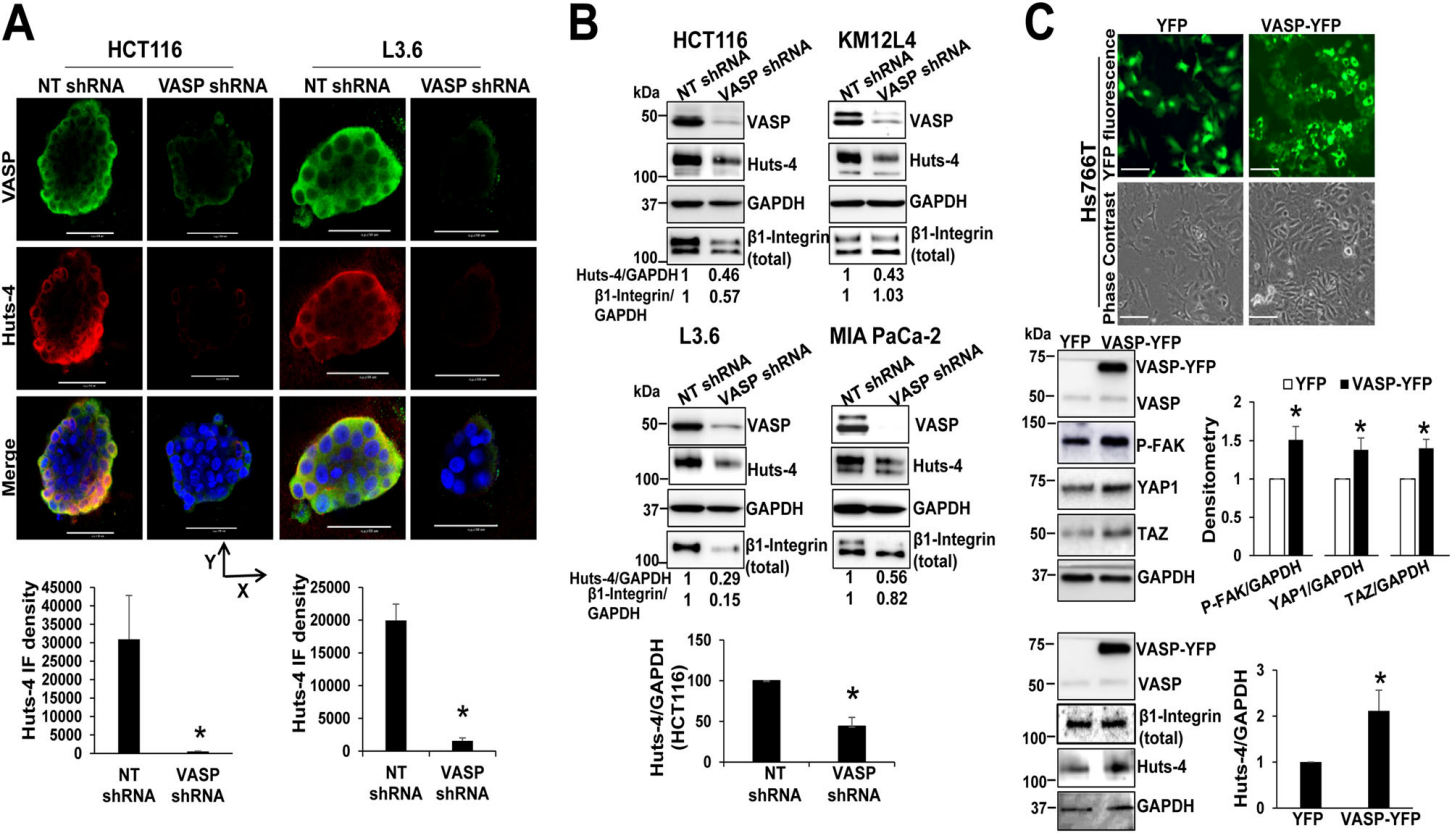
VASP knockdown reduces ECM-mediated β1-integrin activation. **a** Double IF using anti-VASP and Huts-4 (recognizing active β1-integrin) revealed that VASP and Huts-4 colocalized at the outer cellular layers of cancer spheroids and VASP knockdown reduced Huts-4 signals. Representative images of HCT116 and L3.6 cancer spheroids viewed from the top are shown. Quantitative IF data are shown on the bottom. Bar, 100 µm. *, *p* < 0.05, by t-test, *n* = 6, 4 for HCT116 spheroids and *n* = 6, 5 for L3.6 speroids. **b** Cancer spheroids were harvested for WB for Huts-4 under a non-reducing SDS-PAGE. VASP knockdown reduced Huts-4 levels. Densitometry data are shown. *p < 0.05 by *t*-test, *n* = 3. **c** Hs766T cells were transduced with YFP or VASP-YFP retroviruses. Upper, expression of YFP or VASP–YFP fusion protein was detected by fluorescence microscopy. Bar, 100 µm. Lower, WB revealed that VASP-YFP increased *P*-FAK, YAP1/TAZ and active β1-integrin (Huts-4) levels. *p < 0.05, by *t*-test, *n* = 3 repeats. Samples derived from the same experiment and gels/blots were processed in parallel. Error bar: S.D

To test if VASP knockdown suppressed ECM-mediated β1-integrin activation thereby reducing *p–*FAK levels, we collected cancer spheroids and performed double immunofluorescence (IF) using anti–VASP and Huts-4 antibody selectively recognizing β1-integrin in an active conformation.^25^ In HCT116, KM12L4, and L3.6 cell lines, VASP and Huts-4 colocalized at the outer cellular layers of the spheroids (IF images of two cell lines are shown in Fig. 3a and another in Suppl. Figure 1b). Noteworthy, Huts-4 IF signals were dramatically reduced in VASP knockdown spheroids as compared to control spheroids (Fig. 3a and Suppl. Figure 1b, red channel, *p* < 0.05 by *t*-test). In addition, WB confirmed that Huts-4 protein levels of spheroids, in which VASP was knocked-down, were consistently reduced as compared to those of control spheroids (Fig. 3b, quantitative data of HCT116 spheroids are shown, *p* < 0.05 by *t*-test, *n* = 3). The effect of VASP knockdown on the total β1-integrin level, however, was not consistent; the total β1-integrin levels of HCT116, L3.6, and MIAPaCa-1 spheroids were reduced by VASP knockdown and that of KM12L4 spheroids was not (Fig. 3b). In addition to 3D culture, we also analyzed β1-integrin activation at the single-cell level by plating cancer cells onto collage I-coated plates for Huts–4 staining. VASP is a focal adhesion protein that labels focal adhesions of the cell.^26^ In control cells, VASP (green) and Huts-4 (red) colocalized to the peripheral focal adhesions (yellow) (Suppl. Figure 2a). In VASP knockdown cells, Huts-4 IF signals at the peripheral regions were significantly reduced (Suppl. Figure 2a, *p* < 0.05 by *t*-test, *n* = 20). Together, our data support that VASP is required for ECM-mediated activation of β1-integrin and the β1-integrin-FAK signaling.

### Overexpression of VASP promotes β1-integrin-FAK signaling and YAP1/TAZ protein abundance

To understand the role of VASP overexpression in β1-integrin-FAK signaling and YAP1/TAZ, we used WB to quantitate VASP protein levels in multiple cell lines and found that Hs766T (human pancreatic carcinoma cell line) and SW620 (human colorectal adenocarcinoma cell line) express relatively low levels of VASP (Suppl. Figure 2b). Next we transduced Hs766T cells with retroviruses encoding either YFP or VASP-YFP and seeded the cells on Matrigel to induce 3D spheroids. Overexpression of YFP and VASP-YFP fusion proteins was demonstrated by IF and WB (Fig. 3c). As expected, VASP-YFP increased *P*-FAK, YAP1/TAZ and active β1-integrin (Huts-4) levels, as compared to YFP overexpression (Fig. 3c). Thus, VASP overexpression promotes ECM-mediated β1-integrin activation and YAP1/TAZ protein levels in VASP low-expressing cells.

### β1-integrin-FAK signaling promotes YAP1/TAZ protein abundance and targeting the β1-integrin—FAK-YAP1/TAZ signaling inhibits the growth of cancer spheroids

**Fig. 4:**
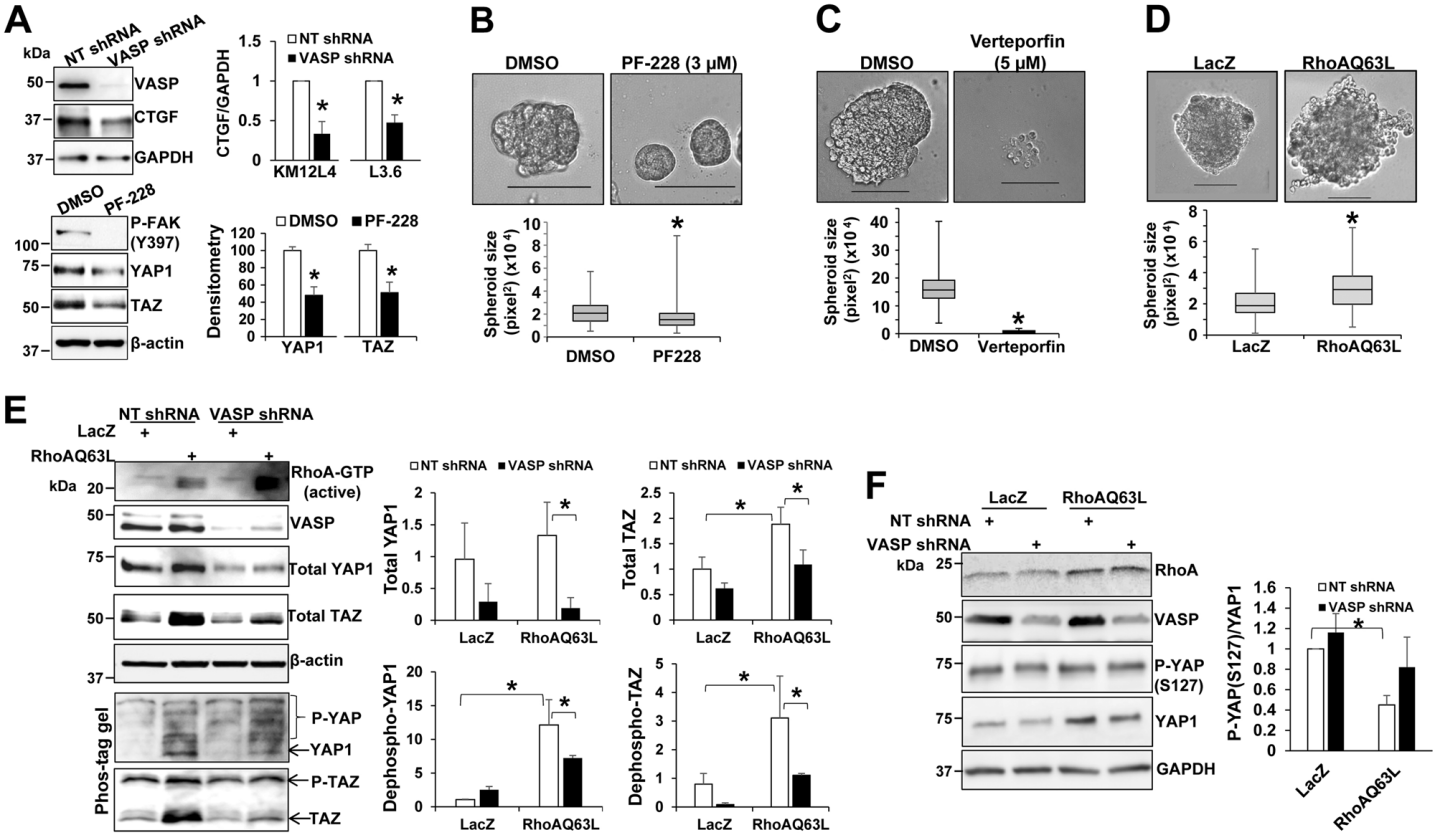
Pharmacologic targeting the β1-integrin-FAK-YAP1/TAZ signaling suppresses cancer spheroids and VASP is required for RhoA-mediated YAP1/TAZ dephosphorylation. **a** Upper, 3D cancer spheroids were harvested for WB for CTGF. VASP knockdown reduced CTGF protein levels in KM12L4 and L3.6 cells. *p < 0.05 by *t*-test, *n* = 3 repeats. Lower, cells on Matrigel were treated with PF–228 (3 μM) and collected for WB. PF-228 reduced YAP1/TAZ protein levels. *p < 0.05 by *t*-test, *n* = 3 repeats. Samples derived from the same experiment and gels/blots were processed in parallel. **b**, **c** PF-228 (3 μM) or Verteporfin (5 μM) reduced the size of L3.6 spheroids on Matrigel. *p < 0.05 by *t*-test; *n* > 50 per group. Bar: 100 µm. **d** HCT116 cells expressing LacZ (control) or RhoAQ63L were seeded on Matrigel to induce cancer spheroids. Overexpression of RhoAQ63L increased the size of cancer spheroids. *p < 0.05 by *t*-test; *n* > 50 per group. **e** Control and VASP knockdown cancer spheroids were harvested for regular WB and Phos-tag^TM^ gel-based WB. RhoAQ63L increased YAP1/TAZ protein levels and YAP1/TAZ dephosphorylation in control cells and these RhoAQ63L effects on YAP1/TAZ were abrogated by VASP knockdown. Densitometry data are shown on the right. *p < 0.05 by ANOVA, *n* = 3. **f** Control and VASP knockdown cancer spheroids were harvested for WB using anti-*P*-YAP(S127) and YAP1. RhoAQ63L reduced the ratio of *P*-YAP(S127) to YAP1 and this effect of RhoA on YAP1 dephosphorylation was partially reversed by VASP knockdown. *p < 0.05 by ANOVA, *n* = 3. Samples derived from the same experiment and gels/blots were processed in parallel. Error bar: S.D

As shown in Fig. 2c, YAP1/TAZ protein levels, downstream targets of the Hippo signaling pathway, were also reduced by VASP knockdown in 3D culture model. This observation is significant since YAP1/TAZ transcriptional coactivators control organ size, regeneration, and tumorigenesis, which is also a key for cancer metastatic growth.^27^ To further strengthen this finding, we performed WB for CTGF, a direct transcriptional target of YAP1/TAZ^19^ and found that VASP knockdown indeed suppressed CTGF protein levels in both KM12L4 and L3.6 cells (Fig. 4a, upper). Next, we tested if YAP1/TAZ were indeed bonafide downstream targets of the β1-integrin-FAK signaling by treating cancer cells with a FAK inhibitor, PF-228, to inhibit auto-phosphorylation of Tyrosine 397 of this kinase. As revealed by Fig. 4a, PF-228 significantly reduced YAP1/TAZ protein levels in cells on Matrigel. These data support that the β1-integrin-FAK signaling pathway indeed promotes YAP1/TAZ protein levels and that VASP is required for ECM-mediated activation of the β1-integrin-FAK-YAP1/TAZ signaling in 3D culture.

To further validate the role of the β1-integrin-FAK-YAP1/TAZ signaling axis in cancer cell survival and growth on ECM, we treated cancer cells with pharmacological inhibitors PF-228 or verteporfin to target against FAK or YAP1 respectively. Quantitative data revealed that each inhibitor reduced the size of cancer spheroids. The median area of spheroids was 20,723 pixel^2^ in control group and reduced to 15,185 pixel^2^ in PF-228-treated group (Fig. 4b, *p* < 0.05 by *t*-test, *n* > 50 spheroids per group) and the median area of spheroids was 157,204 pixel^2^ in control group but reduced to 7228 pixel^2^ in verteporfin-treated group (Fig. 4c, *p* < 0.05 by *t*-test, *n* > 50 spheroids per group). Since VASP knockdown also reduced *P*-Src (Fig. 2c), we treated cells with PP2 to inhibit Src kinase and found that PP2 at 5 or 10 µM significantly reduced the size of cancer spheroids (Suppl. Figure 3a, *p* < 0.05 by AVOVA, *n* > 50 spheroids per group). Thus, the β1-integrin-FAK-YAP1/TAZ signaling axis is a key pro-survival and proliferative factor for cancer cells on ECM.

### RhoA-mediated dephosphorylation and stabilization of YAP1/TAZ requires VASP

Since F-actin or RhoA regulates dephosphorylation, nuclear targeting, and stability of YAP1/TAZ,^28–32^ we speculated that VASP may regulate YAP1/TAZ by additional mechanims, separated from its action on β1-integrin activation. To search for a putative additional mechanism, we focused on RhoA since it is a downstream effector protein of the β1-integrin/FAK and also a signaling intermediate of other cascades such as G-protein-coupled receptors (GPCRs)-mediated signaling.^31,33^ For instance, lysophosphatidic acid (LPA) or sphingosine-1-phosphate (S1P) promotes dephosphorylation and protein stability of YAP1/TAZ by activating RhoA.^31^ Based upon these, we introduced a constitutively active form of RhoA, namely RhoAQ63L,^31^ into cancer cells to test if VASP influenced RhoA-induced activation of YAP1/TAZ. As shown in Fig. 4d, overexpression of RhoAQ63L led to larger spheroids in 3D culture (*p* < 0.05 by *t*-test, *n* > 50 spheroids per group). WB revealed that RhoAQ63L increased YAP1/TAZ protein levels and this effect of RhoA was abrogated by VASP knockdown (Fig. 4e, *p* < 0.05 by ANOVA, *n* = 3). Additionally, Phos-tag^TM^-based WB showed that RhoAQ63L reduced YAP1/TAZ phosphorylation and this effect of RhoA on YAP1/TAZ dephosphorylation was abrogated by VASP knockdown (Fig. 4e, *p* < 0.05 by ANOVA, *n* = 3). Consistently, WB with a YAP phospho-specific antibody (*P*-YAP(S127)) demonstrated that RhoAQ63L reduced the ratio of *P*-YAP(127) to YAP1 and this effect of RhoAQ63L on YAP1 dephosphorylation was partially reversed by VASP knockdown (Fig. 4f, *p* < 0.05 by ANOVA, *n* = 3).

Together, these data support that RhoA promotes YAP1/TAZ dephosphorylation and YAP1/TAZ stability by a VASP-dependent mechanism and that VASP regulates the β1-integrin-RhoA-YAP1/TAZ signaling pathway by at least two mechanisms: (1) promoting β1-integrin activation and (2) promoting YAP1/TAZ dephosphorylation to enhance YAP1/TAZ protein abundance at the downstream of RhoA.

Since tumor microenvironmental LPA or S1P activates GPCRs-RhoA-YAP1/TAZ signaling to promote tumorigenesis,^31^ we hypothesized that VASP may play a role in GPCR-signaling in GI cancer cells. As shown in Suppl. Figure 3b, LPA or S1P stimulation indeed induced a time-dependent upregulation of YAP1/TAZ in control cells but not in VASP knockdown cells. Although these data cannot tell us precisely whether VASP modulates YAP1/TAZ at downstream or upstream of RhoA, the data are of clinical significance given the high relevance of the GPCR-RhoA-YAP1/TAZ signaling cascade in cancer progression and metastasis.

### VASP knockdown suppresses the β1-integrin-YAP1/TAZ pathway and liver metastasis in preclinical mouse models

**Fig. 5:**
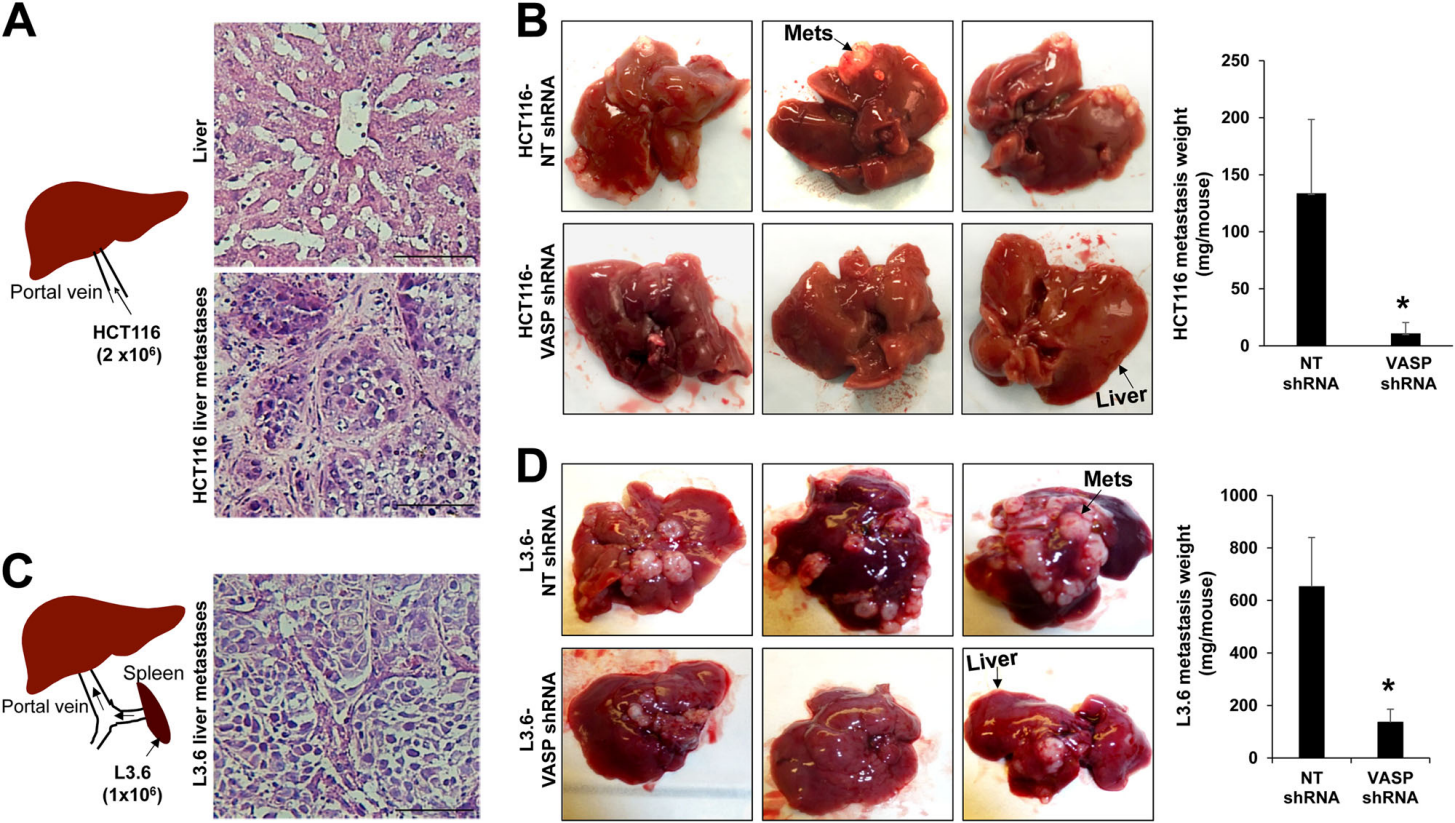
VASP knockdown suppresses liver metastatic growth in mice. **a** Depiction of portal vein injection of HCT116 cells into female SCID mice. H & E staining of the liver and HCT116 liver metastases is shown. Bar, 100 µm. **b** HCT116 cells expressing NT shRNA (control) or VASP shRNA were injected and VASP knockdown reduced HCT116 liver metastases in mice. *p < 0.05 by Mann–Whitney U test, *n* = 9, 10. Error bar: S.E.M. **c** Depiction of intrasplenic injection of L3.6 cells into male SCIDs. H & E staining of L3.6 liver metastases is shown. Bar, 100 µm. **d** L3.6 cancer cells expressing NT shRNA or VASP shRNA were injected and VASP knockdown suppressed L3.6 liver metastases in mice. *p < 0.05 by Mann-Whitney U test, *n* = 5, 7. Error bar: S.E.M

We next used two experimental liver metastasis mouse models to validate VASP as a treatment target for liver metastasis: (1) implantation of HCT116 cells into the liver of female SCID mice by portal vein jection (Fig. 5a)^34,35^ and (2) injection of L3.6 cancer cells into the spleen of male SCID mice (Fig. 5c).^36^ Both models have led to similar results (Fig. 5b, d). The average weight of HCT116 liver metastasis per mouse was 133.7 ± 64.8 mg in control group and reduced to 10.9 ± 9.5 mg in VASP knockdown group (*p* < 0.05 by Mann-Whitney U test, *n* = 9, 10) (Fig. 5b). The average weight of L3.6 liver metastasis per mouse was 652 ± 188 mg in control group and reduced to 135.9 ± 50 mg in VASP knockdown group (*p* < 0.05 by Mann-Whitney U test, *n* = 5, 7) (Fig. 5d). Thus, targeting VASP of cancer cells significantly suppressed liver metastatic growth in both male and female mice.

**Fig. 6:**
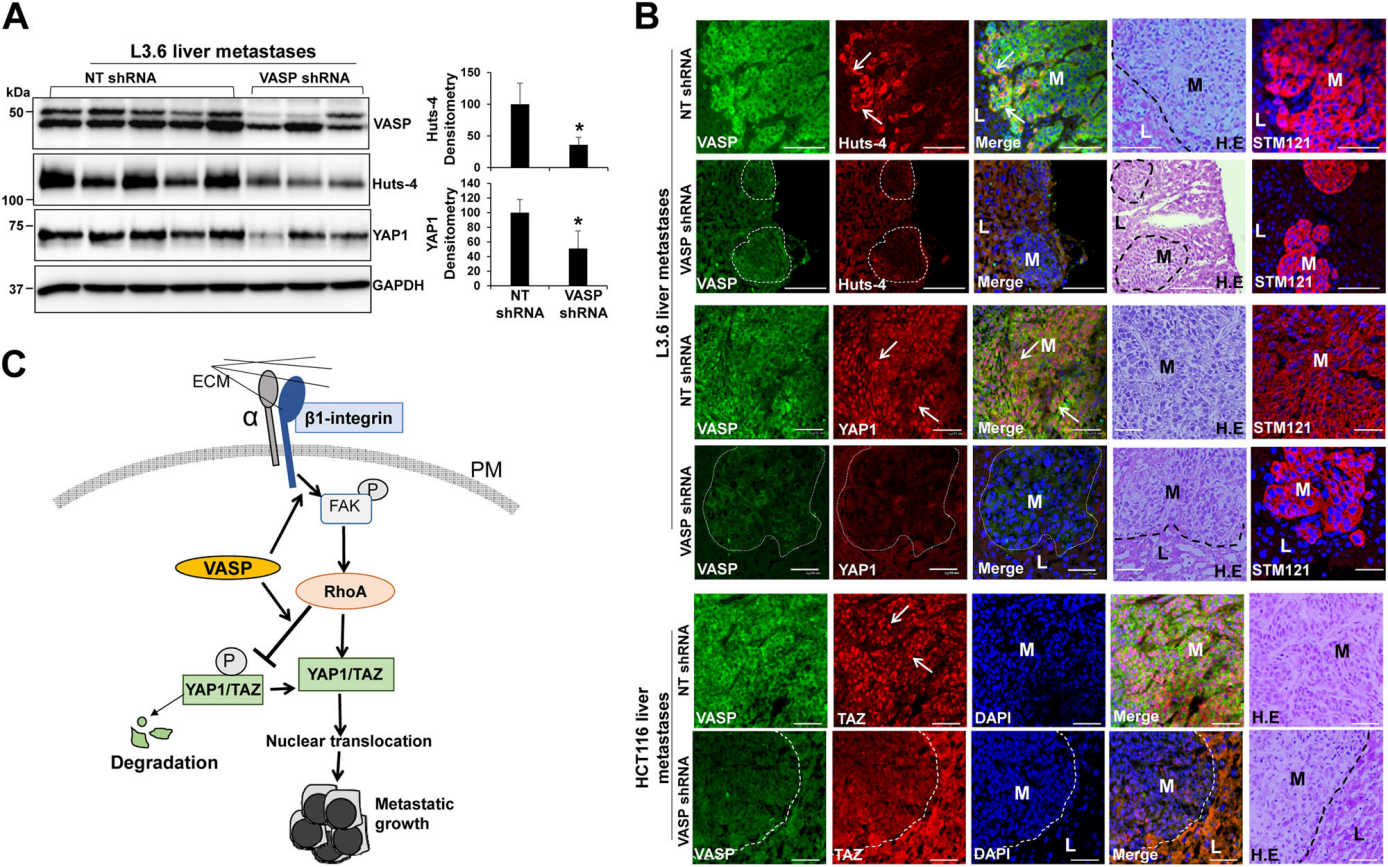
VASP knockdown suppresses β1-integrin activation and YAP1/TAZ protein levels of liver metastatic cells in mice. **a** L3.6 liver metastases were isolated for WB. VASP knockdown reduced Huts-4 and YAP1 levels of metastatic cells as compared to control metastatic cells. *p < 0.05 by *t*-test. *n* = 5, 3. (3 metastases were recovered from VASP knockdown group due to small tumor sizes). Samples derived from the same experiment and gels/blots were processed in parallel. Error bar: S.D. **b** Double IF for VASP/Huts-4 (rows 1, 2), VASP/YAP1 (rows 3, 4), and VASP/TAZ (rows 5, 6) was performed with liver biopsies. L3.6 liver metastases were identified by STEM121 IF. Cell nuclei were counterstained by DAPI. VASP knockdown markedly reduced IF signals of Huts-4, YAP1 and TAZ of metastatic cells as compared to control metastatic cells. L: liver; M: metastases. Bar, 50 µm. **c** VASP promotes cancer cells to colonize the liver by regulating ECM-mediated β1-integrin-FAK-RhoA–YAP1/TAZ signaling. VASP promotes this signaling pathway by two mechanisms: (1) promoting β1–integrin activation and (2) inducing YAP1/TAZ dephosphorylation at downstream of RhoA to enhance YAP1/TAZ protein abundance. PM plasma membrane, P phosphoryl group

HCT116 liver metastases we obtained were much less than L3.6 liver metastases, which prevented us from using them for WB. So we used L3.6 liver metastases to test if VASP knockdown indeed influenced β1-integrin activation and YAP1/TAZ of metastastic cells. For WB, five liver metastases were recovered from control and three were recoved from VASP knockdown group due to small tumor sizes in this group. As revealed by WB for Huts-4 and YAP1, VASP knockdown significantly reduced the protein levels of Huts-4 and YAP1 of liver metastatic cells (Fig. 6a, *p* < 0.05 by *t*-test, *n* = 5, 3). Next we performed IF with liver biopsies of mice. As shown in Fig. 6b, STEM121 antibody, recognizing cells with a human origin,^35^ labeled L3.6 metastatic cancer cells in the liver of mice (column 5). In control metastases, Huts–4 IF signals were strong in the border cells of the tumor mass where cancer invasion of the surrounding liver tissues occurred (row 1, white arrows). In contrast, Huts-4 IF signals were almost absent in VASP knockdown liver metastases (row 2, metastases were circled by dotted lines). Additionally, strong nuclear YAP1 signals were detected in control L3.6 metastatic cells (row 3, white arrows) but not in VASP knockdown L3.6 metastatic cells (row 4). Consistantly, strong nuclear TAZ signals were detected in control HCT116 metastatic cells but not in VASP knockdown HCT116 metastatic cells (Fig. 6b, rows 5 and 6). Thus, targeting VASP of GI cancer cells led to suppression of liver metastasis, β1-integrin activation and YAP1/TAZ abundance of metastatic cells in mice.

## Discussion

Liver metastasis of CRCs and PDACs is a leading cause of cancer-related death due to limited treatment options. Our data, revealing that VASP is upregulated in the majority of CRCs and PDACs with its levels correlated with liver metastasis and reduced patient survival, support an idea that assays against this protein may be developed to serve as a prognostic marker for CRCs and PDACs. Mechanistically, we have shown that VASP activates a β1-integrin-FAK-YAP1/TAZ signaling axis to promote liver metastasis of GI cancers in vitro and in liver metastasis mouse models. In addition, we have identified two mechanisms by which VASP regulates the β1-integrin-FAK-YAP1/TAZ signaling: (1) promoting ECM-induced β1-integrin activation and (2) promoting YAP1/TAZ dephosphorylation at the downstream of RhoA to enhance YAP1/TAZ stability (Fig. 6c). Thus, VASP represents as a therapeutic target for liver metastasis of CRCs and PDACs.

The β1-integrin-FAK signaling is known to promote cancer cells to colonize a distant organ.^7–9^ However, how the β1-integrin-FAK signaling is activated in cancer cells disseminated into the liver, remains poorly understood. In this regard, we have demonstrated, for the first time, that VASP is required for ECM activation of the β1-integrin-FAK signaling and liver metastasis. We have also identified that YAP1/TAZ, transcriptional coactivators, as downstream effector proteins of the β1-integrin-FAK signaling connecting ECM-mediated signaling to cell gene transcription. Furthermore, VASP is required for RhoA-mediated dephosphorylation and stabilization of YAP1/TAZ, at downstream of β1-integrin–FAK signaling. Together, our data provide a novel mechanistic insight into GI cancer survival and metastasis in the liver.

We currently do not know how VASP promotes β1-integrin activation in cancer cells. The binding affinity of integrins for ECM can be low, intermediate, or high and a shift from a low– to high-binding affinity state is termed “integrin activation”,^37^ which is often associated with a conformation change of integrins. Talins or Kindlins promote β1-integrin activation by binding to a NPxY or NxxY motif of the cytoplasmic tail of β1-integrin so as to induce its conformation change.^38,39^ Since Talins and Kindlins directly link to actin cytoskeleton, it is possible that VASP may regulate the binding of Talins or Kindlins to the cytoplasmic tail of β1-integrin. In addition, integrin activation is regulated by its efficient recycling and lysosomal-mediated degradation.^40^ The endocytosed integrins are predominantly in endosomes where they recycle back to the plasma membrane once every 30 min. It has been described that β1–integrin recycles via a long-loop route in an actin-dependent mechanism,^41,42^ suggesting that VASP may be required for the recycling of β1-integrin. Indeed, our data in Fig. 3b revealed that VASP knockdown reduced the total β1-integrin levels in three cancer cell lines, supporting that VASP may regulate protein stability of β1-integrin. Since β1-integrin is degraded by endosome to lysosome sorting and this process is prevented by SNX17, a FERM-like domain-containing sorting nexin,^40,43^ it is possible that VASP may enhance β1-integrin stability by promoting its recycling and suppressing its lysosomal sorting for degradation. Further investigations on these topics may help us gain a full understanding of mechanisms behind β1-integrin activation and the biology of β1-integrin.

We have previously shown that VASP is upregulated in activated hepatic stellate cells (HSCs) (myofibroblasts) of murine and patient colorectal liver metastases.^44^ Additionally, VASP promotes activation of HSCs into tumor-promoting myofibroblasts and potentiates the tumor-promoting effect of activated-HSC/myofibroblasts.^44^ Thus, VASP is a pro-oncogenic factor in both cancer cells and myofibroblasts of the hepatic tumor microenvironment and an ideal therapeutic target for anti-metastasis therapy. Matrine and berberine are two plant extracts from Chinese herbal medicine and they have been shown to bind to VASP to interfere its function so as to inhibit cancer cell proliferation and migration. However, their mechanisms of action and their inhibitory specificity on VASP require further investigation. Indeed, VASP binds to G-actin through its EVH2 domain to promote F-actin assembly, providing us an opportunity to design and develop specific inhibitors to target the actin polymerization activity of VASP. In summary, VASP is a therapeutic target for liver metastasis of GI cancers and our future studies need to focus on novel approaches by which we can screen, identify, and develop compounds to target VASP of both cancer cells and stromal cells.

## Materials and methods

### Cell lines, antibodies, and reagents

KM12L4, HCT116, HT29, MIA PaCa-1, and Hs766T cells were purchased from ATCC (Manassas, VA). L3.6 cells were obtained from Dr. Raul Urrutia at Mayo Clinic.^36^ They were authenticated by short tandem repeat (STR) DNA profiling by Genetica DNA Laboratories in Spring 2017. Cells were routinely tested for mycoplasma and free of infection for experiments.

Antibodies: anti-VASP (610447; BD Transduction Laboratories), Anti-VASP (rabbit polyclonal) (made by Dr. Daniel Billadeau), anti-*P*-FAK (Y397) (44–624G; Thermo Fisher Scientific), anti-FAK (3285; Cell Signaling Technology), anti-Src (36D10; Cell Signaling Technology), anti-*P*-Src (Y416) (D49G4) (6943; Cell Signaling Technology), anti-*P*-p44/42 MAPK (Erk1/2) (Thr202/Tyr204) (D13.14.4E) XP^®^ (4370; Cell Signaling Technology), anti-*P*-Akt (S473) (D9E) XP (4060; Cell Signaling Technology), anti-YAP1 (63.7) (sc-101199; Santa Cruz Biotechnology), anti-*P*-YAP(S127) (D9W2I) (13008; Cell Signaling Technology), anti-TAZ (560235; BD Pharmingen), anti-TAZ (NB600-220; Novus Biological), anti-β1-integrin (9EG7) (550531; BD Biosciences), anti-β1-integrin (mAb13) (552828; BD Biosciences), anti-Huts-4 (MAB2079Z; Chemicon International), anti-RhoA (ARH04, Cytoskeleton), anti-RhoA (26C4) (sc-418; Santa Cruz Biotechnology), anti-β-actin (A5441; Sigma-Aldrich), and anti-GAPDH (G8140; US Biological).

PF-228 (324878) and PP2 (5295730) were purchased from Calbiochem. Verteporfin was from Sigma-Aldrich (SML0534) and Phos-tag^TM^ acrylamide AAL-107 was from the Wako Pure Chemical Industries, Ltd. (304-93521). RhoAQ63L cDNA was obtained from Dr. Debabrata Mukhopadhyay at Mayo Clinic and inserted into the pMMP retroviral vector. The mutation site was confirmed by sequencing. Experiments with pMMP-LacZ were used as controls.

### Matrigel-based 3D culture and cancer spheroid analysis

100 microliter Matrigel^TM^ Matrix (354230, BD Biosciences) were added into a well of an 8-well chamber slide and allowed to solidify in a cell incubator for about 1 h. 2,500 cancer cells suspended in 0.5 ml complete DMEM were seeded in and cultured at 37 °C for about 1 week. Pictures of more than 50 spheroids per group were taken by a Leica DM IRB inverted microscope and quantified using the AxioVision LE64 software. The size of a cancer spheroid is presented as pixel^2^.

### Western blot analysis (WB) of cancer spheroids

A 24-well culture plate pre-coated with Matrigel was used to induce spheroids for WB. The spheroids were lifted by 1 ml cold PBS-EDTA and the replicates within a group were pooled together. After incubation at 4 °C to allow the gel dissolve, spheroids were pelleted and lyzed in RIPA lysis buffer containing PMSF, Na2VO3, NaF, and protease inhibitors (88266, Thermo Fisher Scientific). Protein concentrations were then determined by *DC*^TM^ Protein Assay kit (5000111, Bio-Rad). Active β1-integrin was quantitated by Huts-4 WB after a non-reducing SDS-PAGE^45^ and YAP1/TAZ phosphorylation was determined by Phos-tag^TM^ acrylamide AAL-107-based SDS-PAGE (25 µM) followed by routine blotting and antibody incubation procedures.^35,44,46^ Each experiment was repeated independently for three times.

### Immunofluorescence (IF) and confocal microscopy

Spheroids were first washed with 1 × PBS once, fixed with 4% paraformaldehyde for 15 min, and permeabilized with 0.2% Triton X-100/PBS for 15 min. Subsequent blocking and antibody incubation were done as previously described.^35,44^ Confocal microscopy was performed with a Nikon Eclipse TE2000-E and images were analyzed using the EZ-C1 software (Nikon) and ImageJ software (NIH).

### Transduction of cells with lentiviruses or retroviruses

ShRNA lentiviral constructs, targeting against human VASP, were purchased (NM_003370.3-1294s1c1 and NM_003370.3–1805s1c1; Sigma-Aldrich, St. Louis, MO) and they were validated in one of our prior studies.^44^ Both shRNAs generated consistent results in this study. Lentiviral packaging was done according to protocols recommended by the manufacturer. Retroviruses were generated as we previously described.^26,34^ Cells were transduced with viral supernatant (25–50%) containing polybrene (8 µg/ml) for overnight and harvested 72 hrs later for further experiments.^26,34,35,44^

### RhoA activity assay

RhoA activity was determined by a Pull-down Activation Assay Biochem Kit (BK036, cytoskeleton, Inc.). In brief, 450 µl cell lysate (275 µg total protein) was incubated with 30 µl agarose beads conjugated with Rhotekin-Rho binding domain at 4 °C for 1 hr. The beads were precipitated by centrifugation and RhoA pulled-down was then determined by WB using a RhoA specific antibody, provided by the kit.

### Clinical samples and Kaplan–Meier survival analysis

A total of 63 CRCs and 53 PDACs and corresponding liver metastatic tumors were collected from patients who underwent resection and all tumors were histologically confirmed as CRCs or PDACs. The tumor stages were classified according to the 6th edition tumor–node–metastasis (TNM) classification criteria of the Union for International Cancer Control (UICC). This study enrolled 60 men and 56 women with ages ranging from 33 to 100 years. All tissues resected were fixed with 10% formalin and embedded in paraffin. The patients with evidence of concomitant extrahepatic diseases were excluded from the study. Immunohistochemistry (IHC) with patient samples was approved by the Institutional Review Board (IRB) of St. Louis University, School of Medicine. In brief, tissue arrays or whole tumor sections were used for VASP IHC with VASP immunoreactivity blindly scored as −, +, ++, or +++ by board–certified pathologists.^47^ Additionally, 27 patients with paired primary CRCs and liver biopsies were randomly picked for analyzing a correlation between VASP and CRC liver metastasis.

RNA sequencing data of 150 PDAC patients in the Cancer Genome Atlas (TCGA) and their clinical information were downloaded from the Xena Public Data Hubs (https://xena.ucsc.edu). The log2(*x* + 1) transformed RSEM normalized counts were reported for gene expression levels.^48^ The Kaplan–Meier survival curves were generated based on VASP gene expression levels. The difference between groups was determined by Log-rank test.

### Intrasplenic and portal vein tumor injection mouse models

Animal studies were approved by the Institutional Animal Care and Use Committee (IACUC) of the University of Minnesota. 2-month-old male or female SCID mice were purchased from Charles River Frederick Research Model Facility. They were randomly divided into four groups for tumor implantation. Under a general anesthesia by isoflurane, a 1–2 cm incision was made on the left side of the abdomen of a SCID mouse to expose the spleen or portal vein, where the cells were injected, using a sterile 0.5cc insulin syringe with a permanent needle of 27 G.^34–36^ 2 × 10^6^ HCT116 cells in 100 µl PBS were injected into a portal vein or 1 × 10^6^ L3.6 cells in 33 µl PBS were injected into a spleen of mice. Mice received a post-operative care and monitoring according to the IACUC guidelines. For portal vein injection, *n* = 9, 10 mice survived the surgery and for intrasplenic injection, *n* = 5, 7 mice survived. The resulting liver metastases were subjected to WB and IF analyses blindly by a different investigator.

### Statistical analysis

In vitro data are presented as mean ± S.D. Two-tailed Student’s *t-*test or ANOVA followed by a posthoc test was used to evaluate the difference using the GraphPad Prism 5 software (GraphPad Software, Inc., La Jolla, CA). Tumor data of mice are presented as Mean ± S.E.M. and analyzed by the Mann–Whitney U test. The correlation between VASP and CRC liver metastasis of patients was analyzed by the Fisher’s Exact Test and Kaplan–Meier survival curves were analyzed by Log-rank test. *p* < 0.05 was considered as statistically different.

### Study approval

Experiments with animals were approved by IACUC of the University of Minnesota and experiments with patient samples were approved by IRB of St. Louis University School of Medicine. All methods involving animals and patient biopsies were performed in accordance with relevant regulations and guidelines and written informed consent was obtained from each patient prior to the start of the study.

### Data availability

The authors declare that the data supporting our findings are included in the paper and its supplemental information files.

## Electronic supplementary material

Supplemental Figure legends

Supplemental Figures

Western full blot

## Electronic supplementary material

**Supplementary information** accompanies the paper on the *npj Precision Oncology* website (10.1038/s41698-017-0045-7).

## References

[1] Bouvard D, Pouwels J, De Franceschi N, Ivaska J (2013). Integrin inactivators: balancing cellular functions in vitro and in vivo. Nat. Rev. Mol. Cell Biol..

[2] Brakebusch C, Bouvard D, Stanchi F, Sakai T, Fassler R (2002). Integrins in invasive growth. J. Clin. Invest..

[3] Cance WG (2000). Immunohistochemical analyses of focal adhesion kinase expression in benign and malignant human breast and colon tissues: correlation with preinvasive and invasive phenotypes. Clin. Cancer Res..

[4] Owens LV (1995). Overexpression of the focal adhesion kinase (p125FAK) in invasive human tumors. Cancer Res..

[5] White DE (2004). Targeted disruption of beta1-integrin in a transgenic mouse model of human breast cancer reveals an essential role in mammary tumor induction. Cancer Cell.

[6] Haier J, Nasralla M, Nicolson GL (1999). Different adhesion properties of highly and poorly metastatic HT-29 colon carcinoma cells with extracellular matrix components: role of integrin expression and cytoskeletal components. Br. J. Cancer.

[7] Shibue T, Weinberg RA (2009). Integrin beta1-focal adhesion kinase signaling directs the proliferation of metastatic cancer cells disseminated in the lungs. Proc. Natl. Acad. Sci. USA.

[8] Barkan D, Chambers AF (2011). Beta1-integrin: a potential therapeutic target in the battle against cancer recurrence. Clin. Cancer Res..

[9] Brakebusch C, Fassler R (2005). Beta 1 integrin function in vivo: adhesion, migration and more. Cancer Metastasis Rev..

[10] Krause M, Dent EW, Bear JE, Loureiro JJ, Gertler FB (2003). Ena/VASP proteins: regulators of the actin cytoskeleton and cell migration. Annu. Rev. Cell Dev. Biol..

[11] Trichet L, Sykes C, Plastino J (2008). Relaxing the actin cytoskeleton for adhesion and movement with Ena/VASP. J. Cell Biol..

[12] Bear JE, Gertler FB (2009). Ena/VASP: towards resolving a pointed controversy at the barbed end. J. Cell Sci..

[13] Butt E (1994). cAMP—and cGMP-dependent protein kinase phosphorylation sites of the focal adhesion vasodilator-stimulated phosphoprotein (VASP) in vitro and in intact human platelets. J. Biol. Chem..

[14] Harbeck B, Huttelmaier S, Schluter K, Jockusch BM, Illenberger S (2000). Phosphorylation of the vasodilator-stimulated phosphoprotein regulates its interaction with actin. J. Biol. Chem..

[15] Blume C (2007). AMP-activated protein kinase impairs endothelial actin cytoskeleton assembly by phosphorylating vasodilator-stimulated phosphoprotein. J. Biol. Chem..

[16] Schlegel N (2008). The role of VASP in regulation of cAMP—and Rac 1-mediated endothelial barrier stabilization. Am. J. Physiol. Cell Physiol..

[17] Massberg S (2004). Enhanced in vivo platelet adhesion in vasodilator-stimulated phosphoprotein (VASP)-deficient mice. Blood.

[18] Han G (2008). Positive regulation of migration and invasion by vasodilator-stimulated phosphoprotein via Rac1 pathway in human breast cancer cells. Oncol. Rep..

[19] Zhao B (2008). TEAD mediates YAP-dependent gene induction and growth control. Genes Dev..

[20] Zhao B, Li L, Lei Q, Guan KL (2010). The Hippo-YAP pathway in organ size control and tumorigenesis: an updated version. Genes Dev..

[21] Hao Y, Chun A, Cheung K, Rashidi B, Yang X (2008). Tumor suppressor LATS1 is a negative regulator of oncogene YAP. J. Biol. Chem..

[22] Dupont S (2011). Role of YAP/TAZ in mechanotransduction. Nature.

[23] Piccolo S, Dupont S, Cordenonsi M (2014). The biology of YAP/TAZ: hippo signaling and beyond. Physiol. Rev..

[24] Mantel N (1966). Evaluation of survival data and two new rank order statistics arising in its consideration. Cancer Chemother. Rep..

[25] Luque A (1996). Activated conformations of very late activation integrins detected by a group of antibodies (HUTS) specific for a novel regulatory region (355-425) of the common beta 1 chain. J. Biol. Chem..

[26] Kang N (2010). Focal adhesion assembly in myofibroblasts fosters a microenvironment that promotes tumor growth. Am. J. Pathol..

[27] Moroishi T, Hansen CG, Guan KL (2015). The emerging roles of YAP and TAZ in cancer. Nat. Rev. Cancer.

[28] Wada K, Itoga K, Okano T, Yonemura S, Sasaki H (2011). Hippo pathway regulation by cell morphology and stress fibers. Development.

[29] Kim M (2013). cAMP/PKA signalling reinforces the LATS-YAP pathway to fully suppress YAP in response to actin cytoskeletal changes. EMBO J..

[30] Aragona M (2013). A mechanical checkpoint controls multicellular growth through YAP/TAZ regulation by actin-processing factors. Cell.

[31] Yu FX (2012). Regulation of the Hippo-YAP pathway by G-protein-coupled receptor signaling. Cell.

[32] Zhang Y (2014). CD44 acts through RhoA to regulate YAP signaling. Cell Signal..

[33] Huveneers S, Danen EH (2009). Adhesion signaling—crosstalk between integrins, Src and Rho. J. Cell Sci..

[34] Decker NK (2008). Nitric oxide regulates tumor cell cross-talk with stromal cells in the tumor microenvironment of the liver. Am. J. Pathol..

[35] Liu C (2013). IQGAP1 suppresses TbetaRII-mediated myofibroblastic activation and metastatic growth in liver. J. Clin. Invest..

[36] Bruns CJ, Harbison MT, Kuniyasu H, Eue I, Fidler IJ (1999). In vivo selection and characterization of metastatic variants from human pancreatic adenocarcinoma by using orthotopic implantation in nude mice. Neoplasia.

[37] Moser M, Legate KR, Zent R, Fassler R (2009). The tail of integrins, talin, and kindlins. Science.

[38] Calderwood DA (1999). The Talin head domain binds to integrin beta subunit cytoplasmic tails and regulates integrin activation. J. Biol. Chem..

[39] Ma YQ, Qin J, Wu C, Plow EF (2008). Kindlin-2 (Mig-2): a co-activator of beta3 integrins. J. Cell Biol..

[40] Bottcher RT (2012). Sorting nexin 17 prevents lysosomal degradation of beta1 integrins by binding to the beta1-integrin tail. Nat. Cell Biol..

[41] Powelka AM (2004). Stimulation-dependent recycling of integrin beta1 regulated by ARF6 and Rab11. Traffic.

[42] Puthenveedu MA (2010). Sequence-dependent sorting of recycling proteins by actin-stabilized endosomal microdomains. Cell.

[43] Steinberg F, Heesom KJ, Bass MD, Cullen PJ (2012). SNX17 protects integrins from degradation by sorting between lysosomal and recycling pathways. J. Cell Biol..

[44] Tu K (2014). VASP promotes TGF-beta activation of hepatic stellate cells by regulating Rab11 dependent plasma membrane targeting of TGF-beta receptors. Hepatology.

[45] Paszek MJ (2005). Tensional homeostasis and the malignant phenotype. Cancer Cell.

[46] Zhao B, Li L, Tumaneng K, Wang CY, Guan KL (2010). A coordinated phosphorylation by Lats and CK1 regulates YAP stability through SCF(beta-TRCP). Genes Dev..

[47] Lai JP (2015). Comparison of PAX6 and PAX8 as immunohistochemical markers for pancreatic neuroendocrine tumors. Endocr. Pathol..

[48] Zhang W (2015). Network-based isoform quantification with RNA-Seq. PLoS Comput. Biol..

