# Supplementary material for: Vasodilator-stimulated phosphoprotein promotes liver metastasis of gastrointestinal cancer by activating a β1-integrin-FAK-YAP1/TAZ signaling pathway
Source: NPJ Precis Oncol. 2018 Jan 23;2:2. doi: 10.1038/s41698-017-0045-7 (PMC5871906; doi:10.1038/s41698-017-0045-7)
Supplement: Supplementary file 1 — Supplemental Figure legends [file 41698_2017_45_MOESM1_ESM.pdf]

Supplemental Figure Legends

Suppl. Fig. 1. A. VASP knockdown in HT29 CRC cells significantly reduced the size of cancer spheroids. *, $p<0.05$ by *t*-test, n>50 per group; Bar, 100 µm. Error bar: S.D. **B.** Double IF using anti-VASP and Huts-4 (recognizing active β1-integrin) revealed that VASP and Huts-4 colocalized at the outer cellular layers of cancer spheroids and VASP knockdown reduced Huts-4 signals. Representative images of KM12L4 spheroids viewed from the top are shown. Quantitative IF data shown on the right. Bar, 100 µm. *, $P<0.05$, by t-test, n=6. Error bar: S.D.

Suppl. Fig. 2. A. Cancer cells on collagen I-coated dishes were collected for double IF. In control cells, VASP (green) and Huts-4 (red) were concentrated at the peripheral focal adhesions (yellow). In VASP knockdown cells, Huts-4 IF signals at the cell peripheral regions were significantly reduced. *, $p<0.05$ by *t*-test, n=20. Bar, 20 µm. Error bar: S.D. **B.** 7 human cancer cell lines were subjected to WB to determine VASP protein levels. Densitometry data are shown. Hs766T and SW620 express relatively low levels of VASP protein. Samples derived from the same experiment and gels/blots were processed in parallel. Data were consistent in 2 repeats.

Suppl. Fig. 3. A. Src inhibitor PP2 (5 µM or 10 µM) reduced the size of L3.6 spheroids. *, $P<0.05$ by ANOVA; n>50 per group. Bar: 100 µm. Error bar: S.D. **B.** Cancer cells treated with LPA (lysophosphatidic acid) or S1P (sphingosine-1-phosphate) for different times were collected for WB to quantitate YAP1/TAZ. LPA or S1P stimulation induced time-dependent increases of YAP1/TAZ proteins and this effect of LPA or S1P on YAP1/TAZ was abropgated by VASP knockdown. Data represent consistent results of multiple repeats. Samples derived from the same experiment and gels/blots were processed in parallel.
