# Supplementary figures and images for: Vasodilator-stimulated phosphoprotein promotes liver metastasis of gastrointestinal cancer by activating a β1-integrin-FAK-YAP1/TAZ signaling pathway
Source: NPJ Precis Oncol. 2018 Jan 23;2:2. doi: 10.1038/s41698-017-0045-7 (PMC5871906; doi:10.1038/s41698-017-0045-7)

Suppl. Figure 1

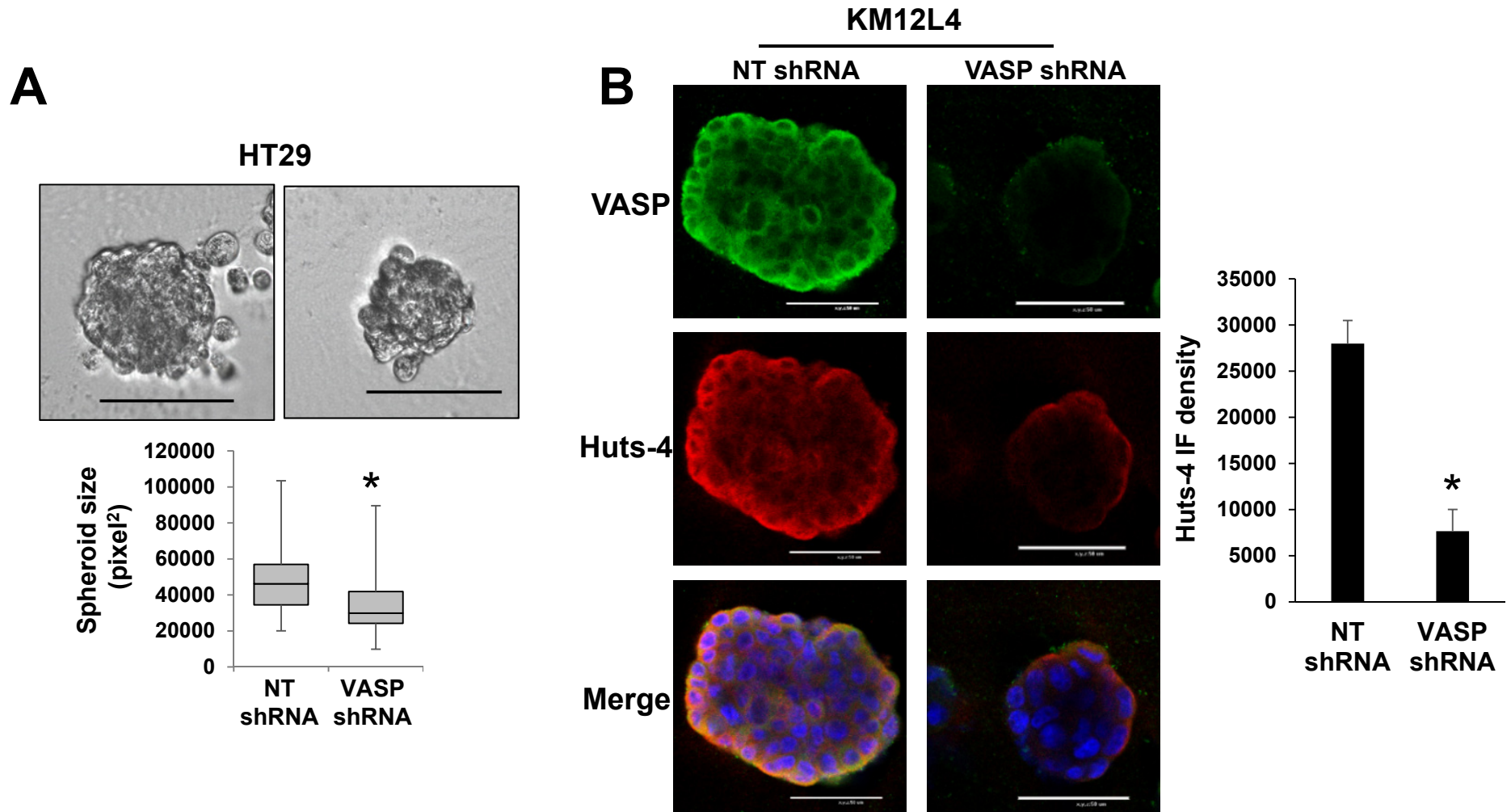

Suppl. Figure 2

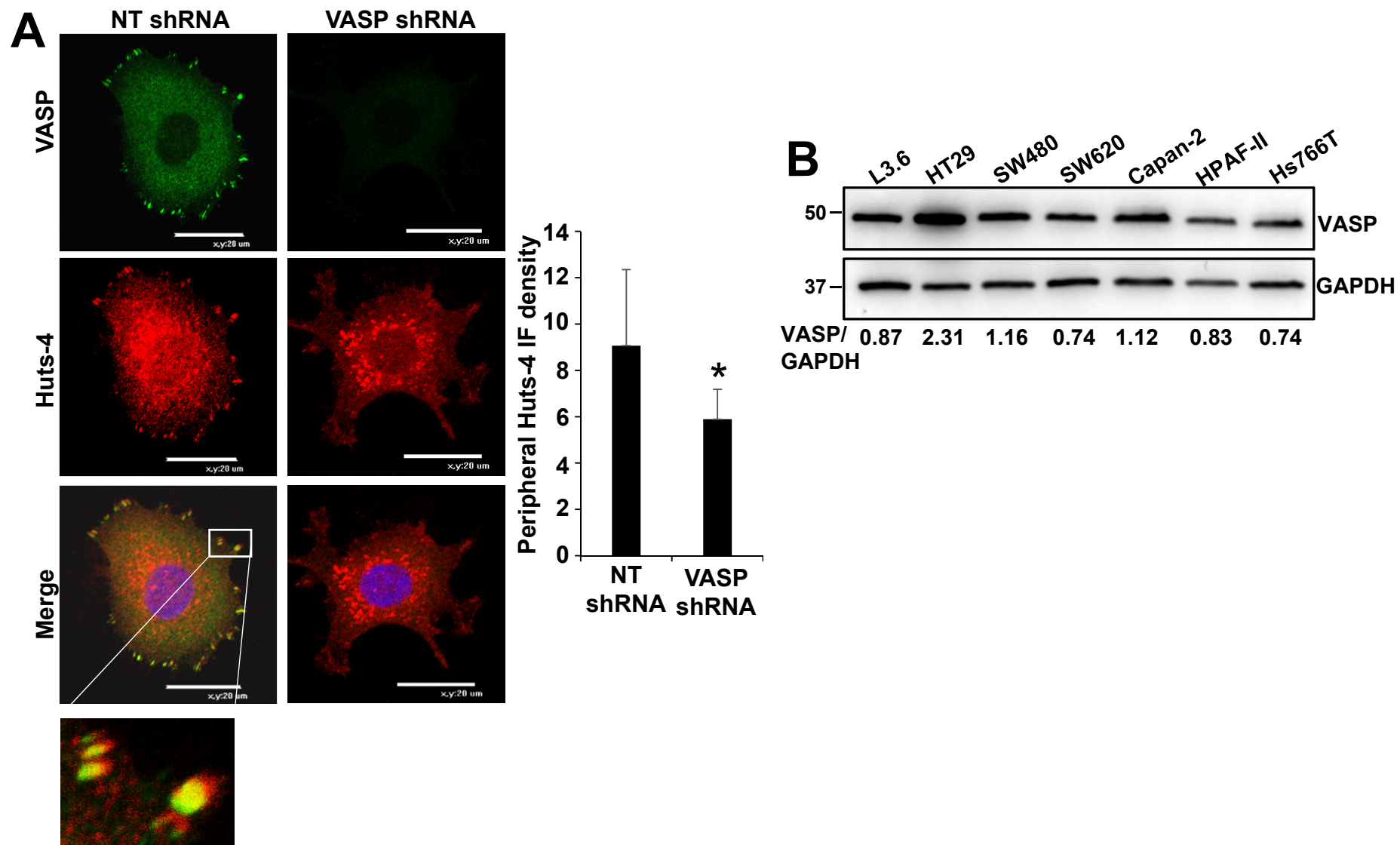

# B

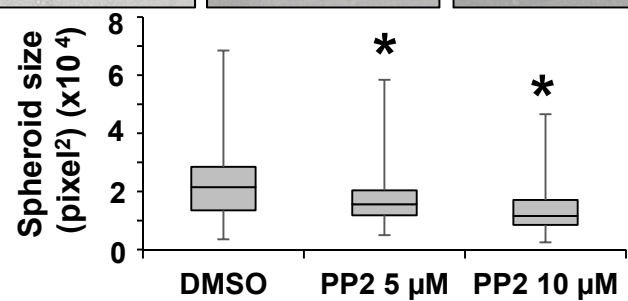

# B

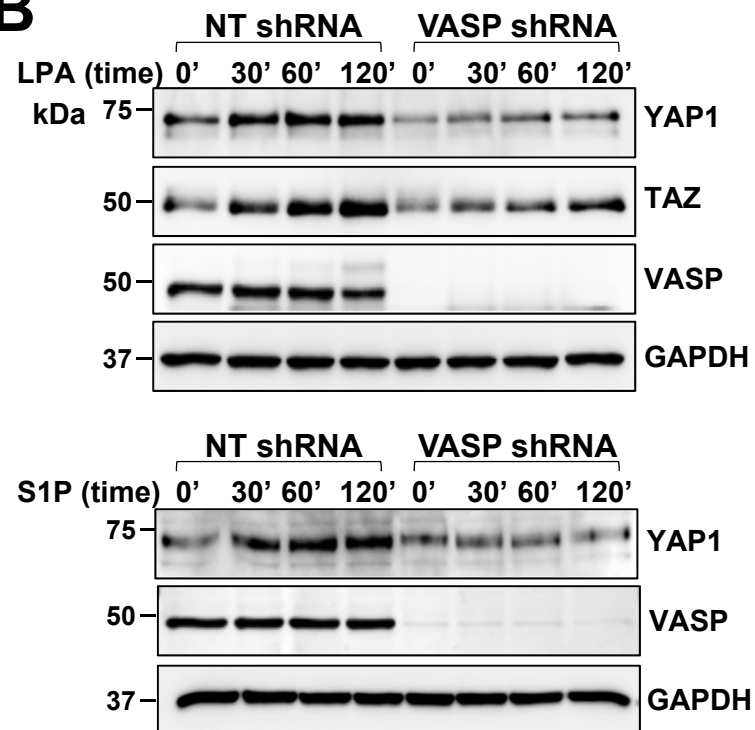

Supplement: Supplementary file 2 — Supplemental Figures [file 41698_2017_45_MOESM2_ESM.pdf]
