# Supplementary material for: Vasodilator-stimulated phosphoprotein promotes liver metastasis of gastrointestinal cancer by activating a β1-integrin-FAK-YAP1/TAZ signaling pathway
Source: NPJ Precis Oncol. 2018 Jan 23;2:2. doi: 10.1038/s41698-017-0045-7 (PMC5871906; doi:10.1038/s41698-017-0045-7)

**Fig.2A**

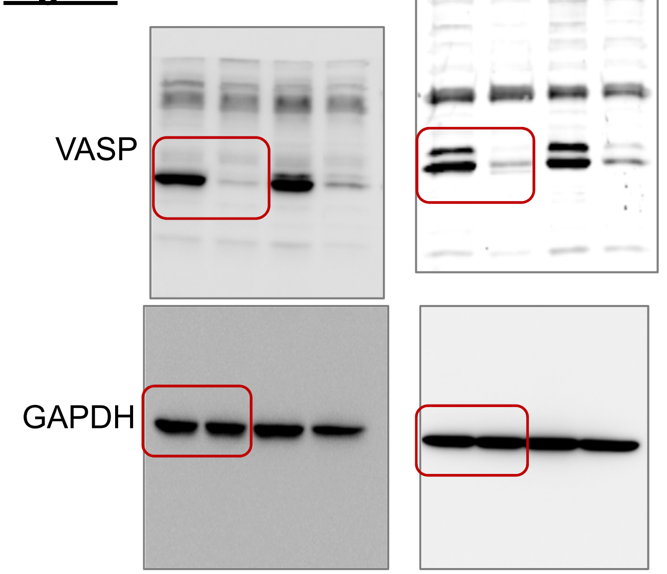


**Fig.2B**

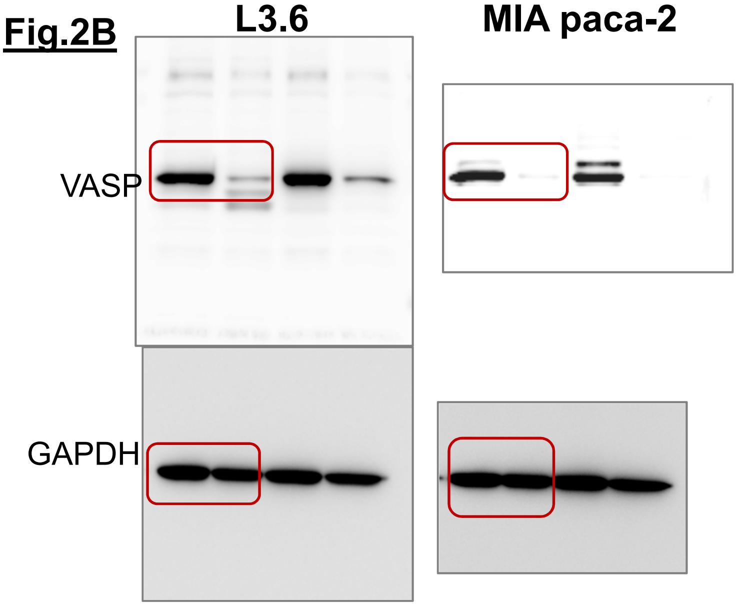


**Fig.2C**

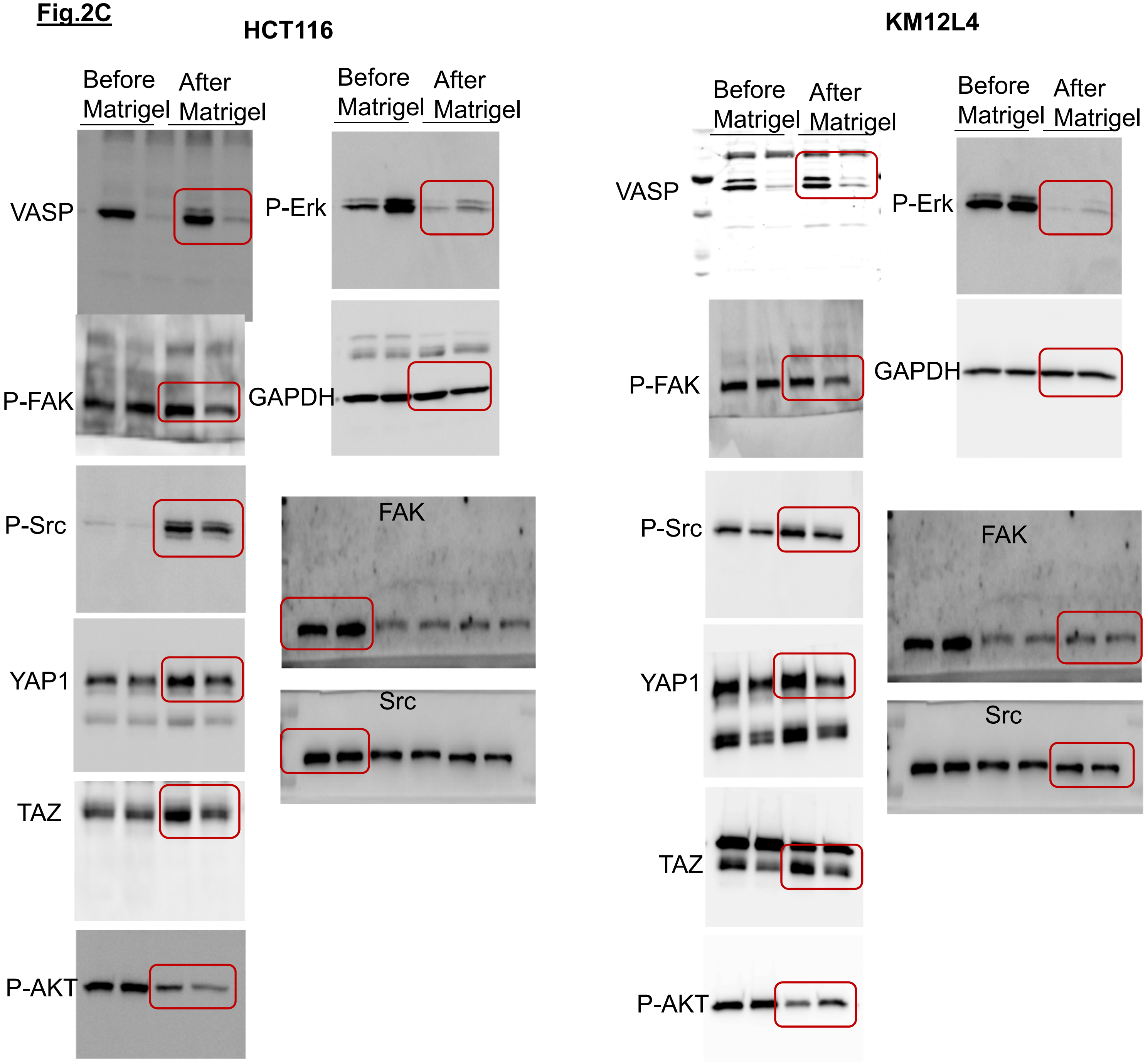

**Fig.2C**

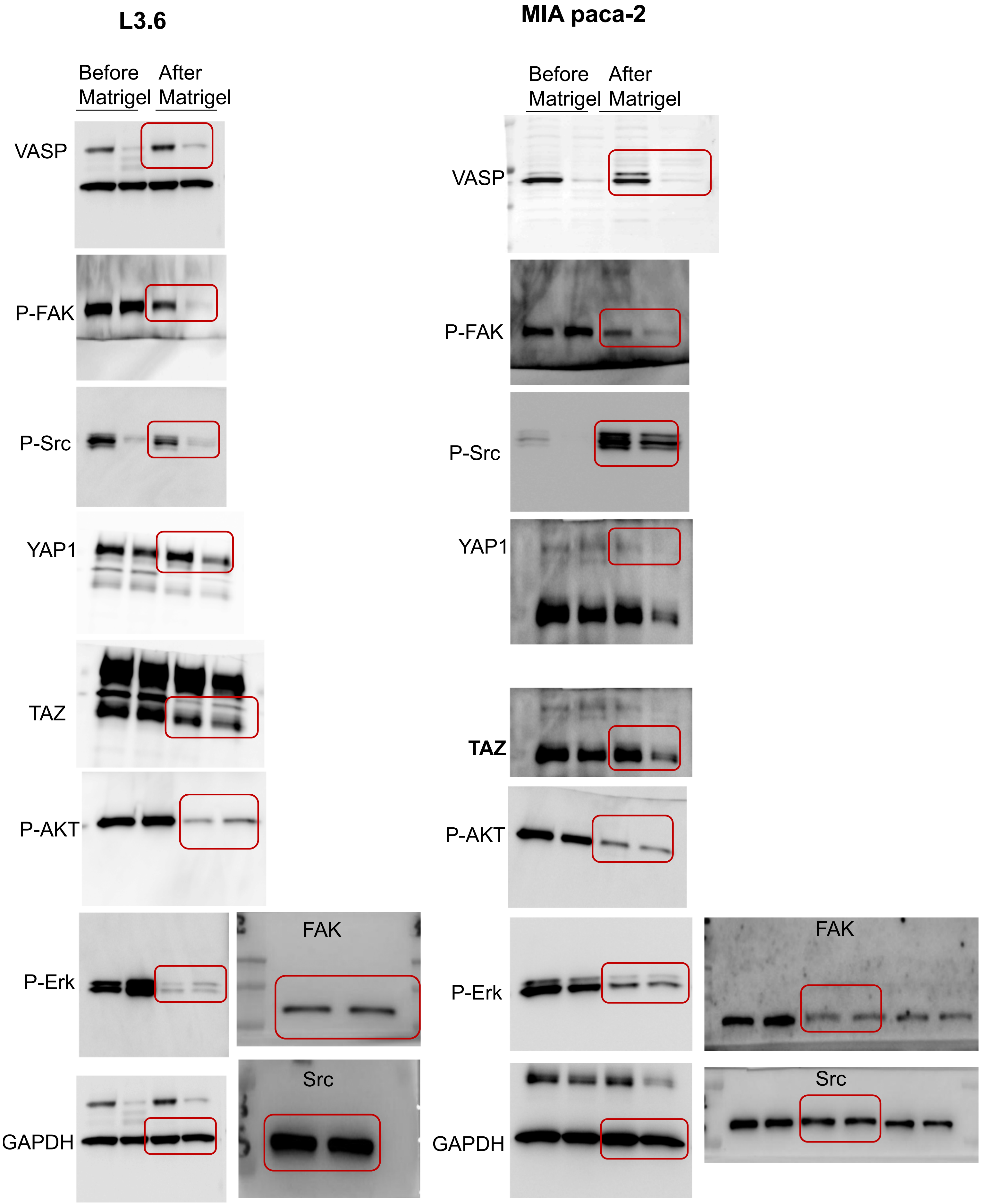

L3.6
MIA paca-2
Before Matrigel
After Matrigel
Before Matrigel
After Matrigel
VASP
P-FAK
P-Src
YAP1
TAZ
P-AKT
P-Erk
FAK
GAPDH
Src
VASP
P-FAK
P-Src
YAP1
TAZ
P-AKT
P-Erk
FAK
GAPDH
Src

Fig.3B

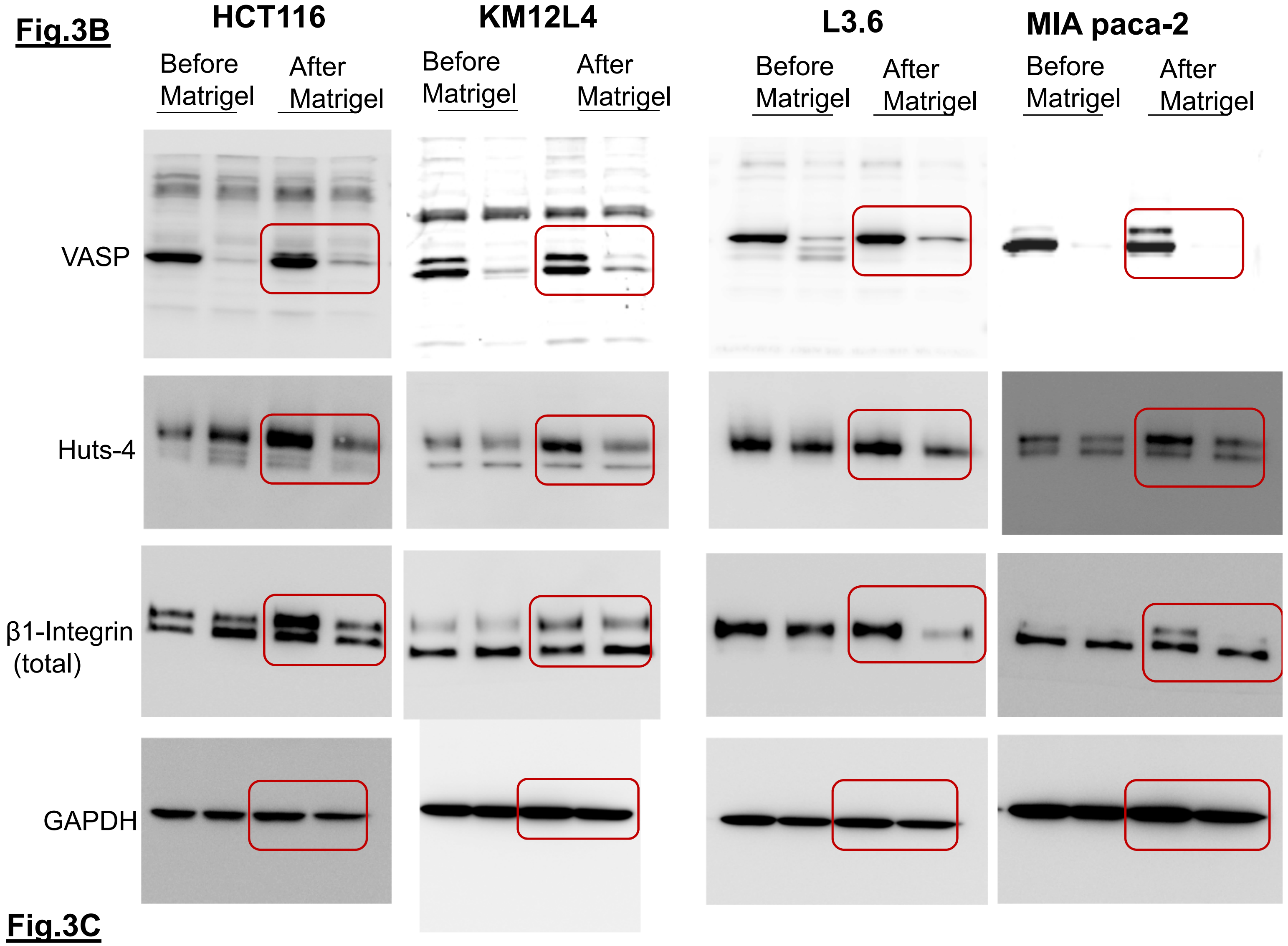
HCT116
KM12L4
L3.6
MIA paca-2
Before Matrigel
After Matrigel
VASP
Huts-4
β1-Integrin (total)
GAPDH

Fig.3C

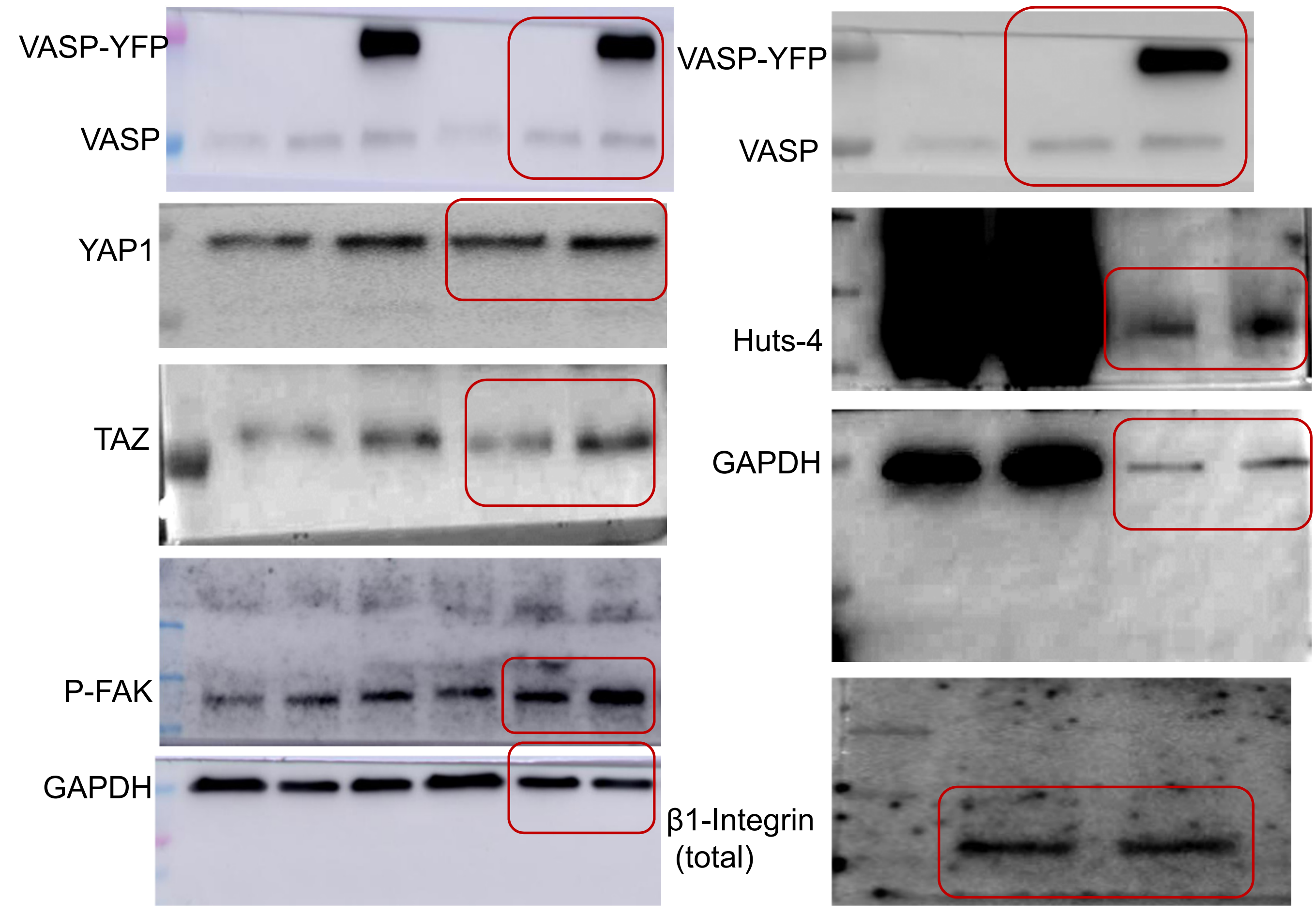
VASP-YFP
VASP
YAP1
TAZ
P-FAK
GAPDH
VASP-YFP
VASP
Huts-4
GAPDH
β1-Integrin (total)

**Fig. 4A top**

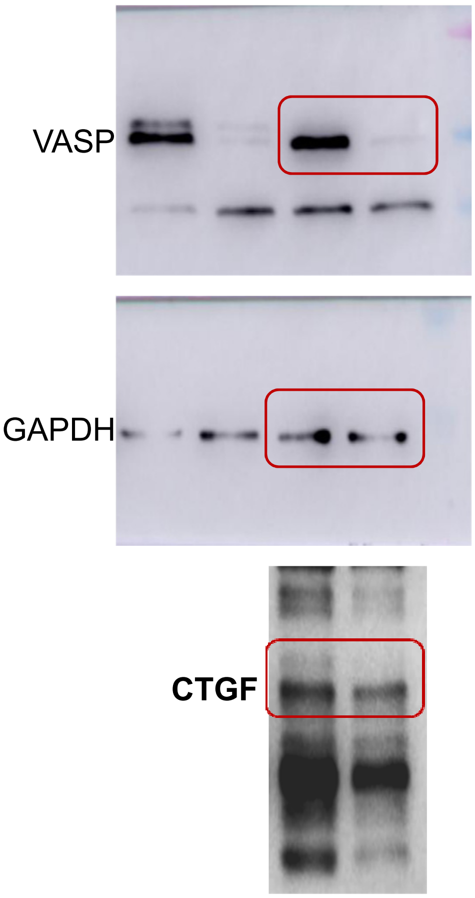


**Fig. 4A bottom**

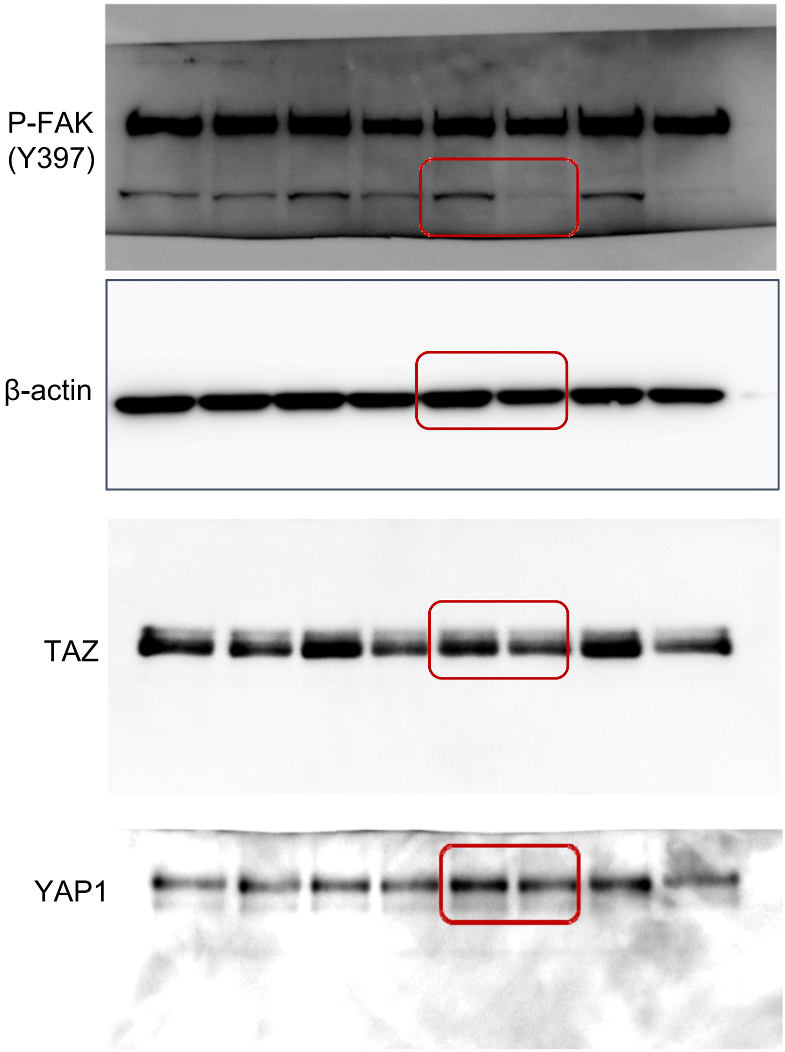


**Fig. 4E Upper**

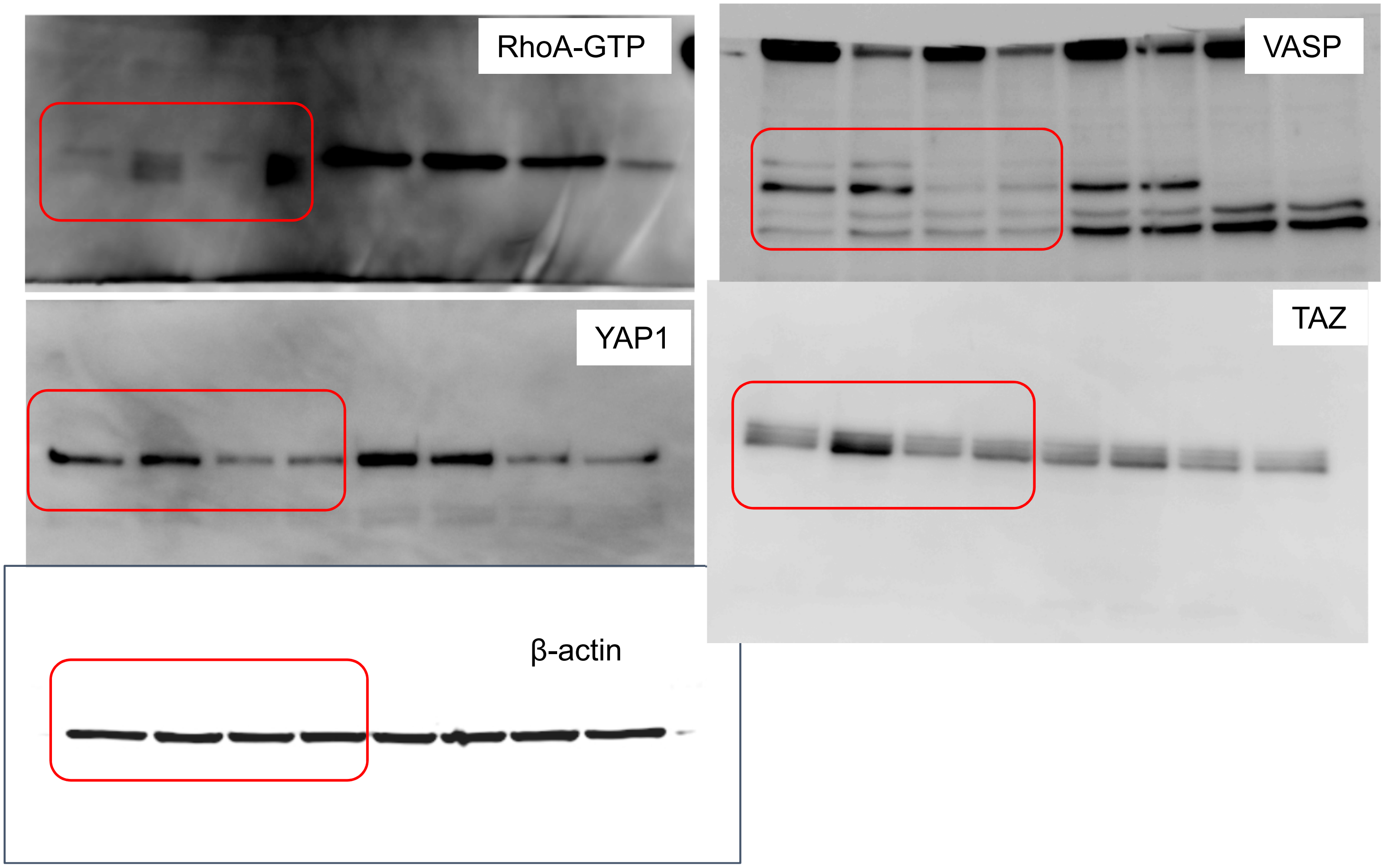

**Fig. 4E lower, phos-tag gel**

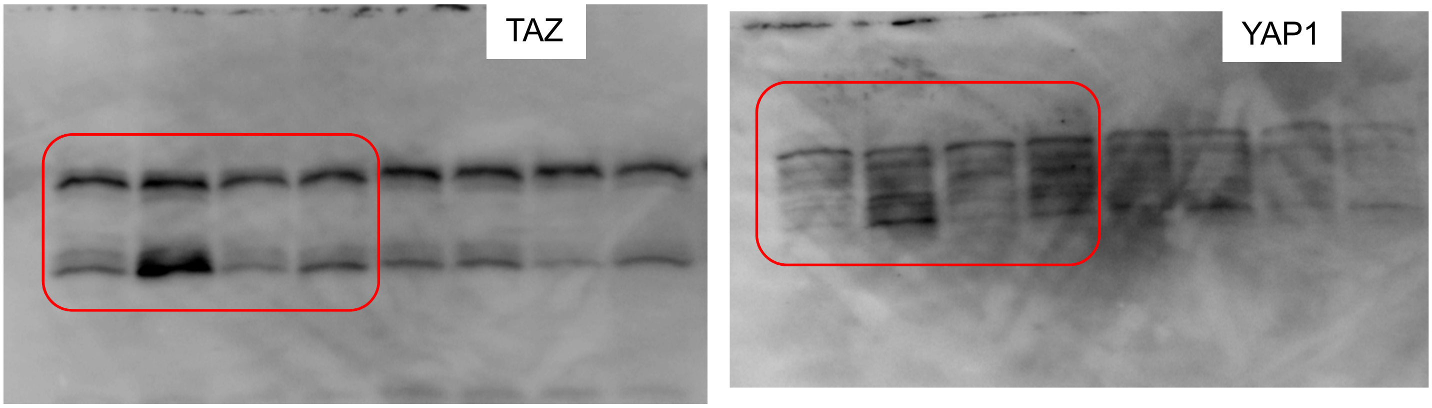


**Fig. 4F**

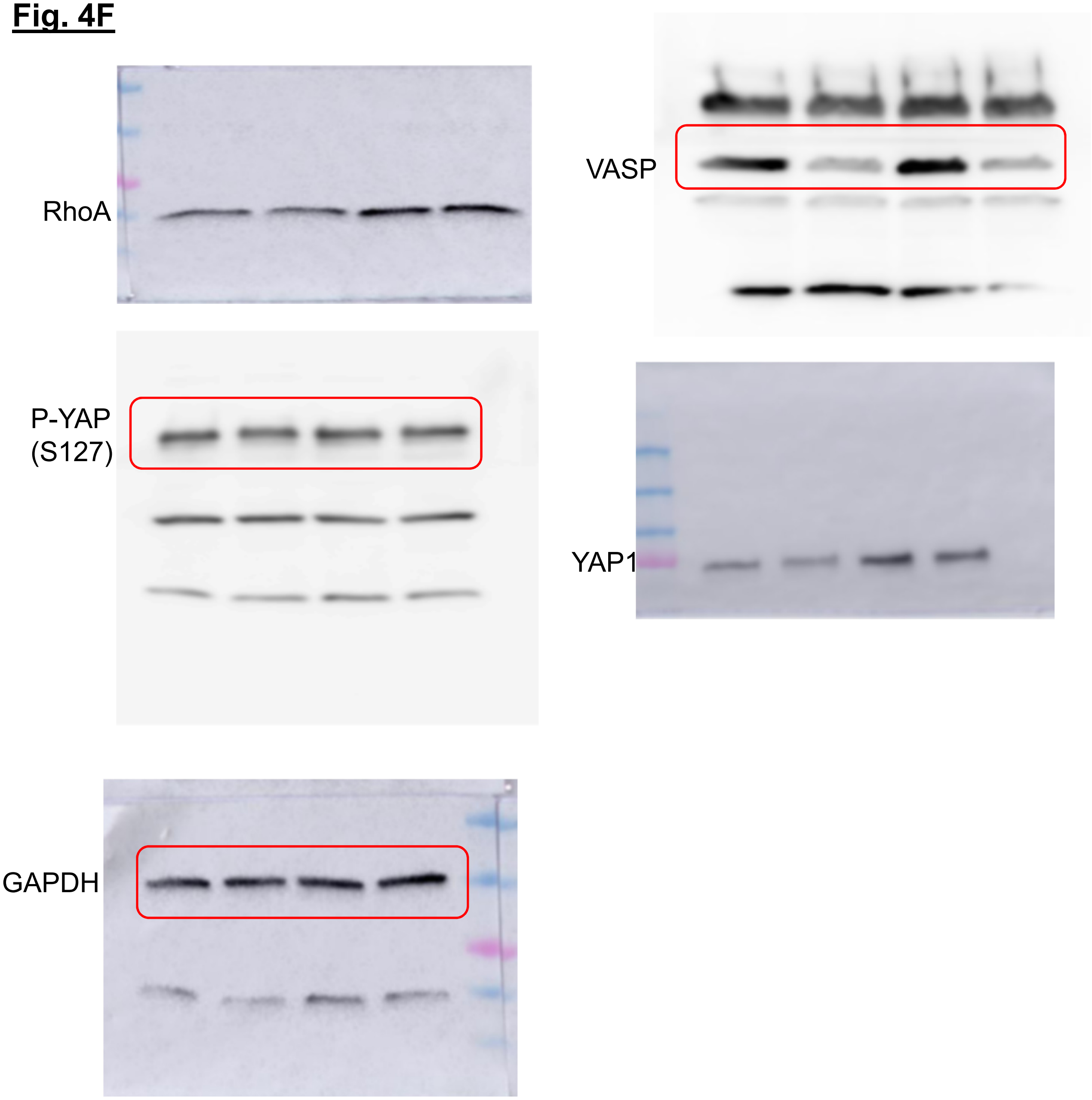

**Fig.6A**

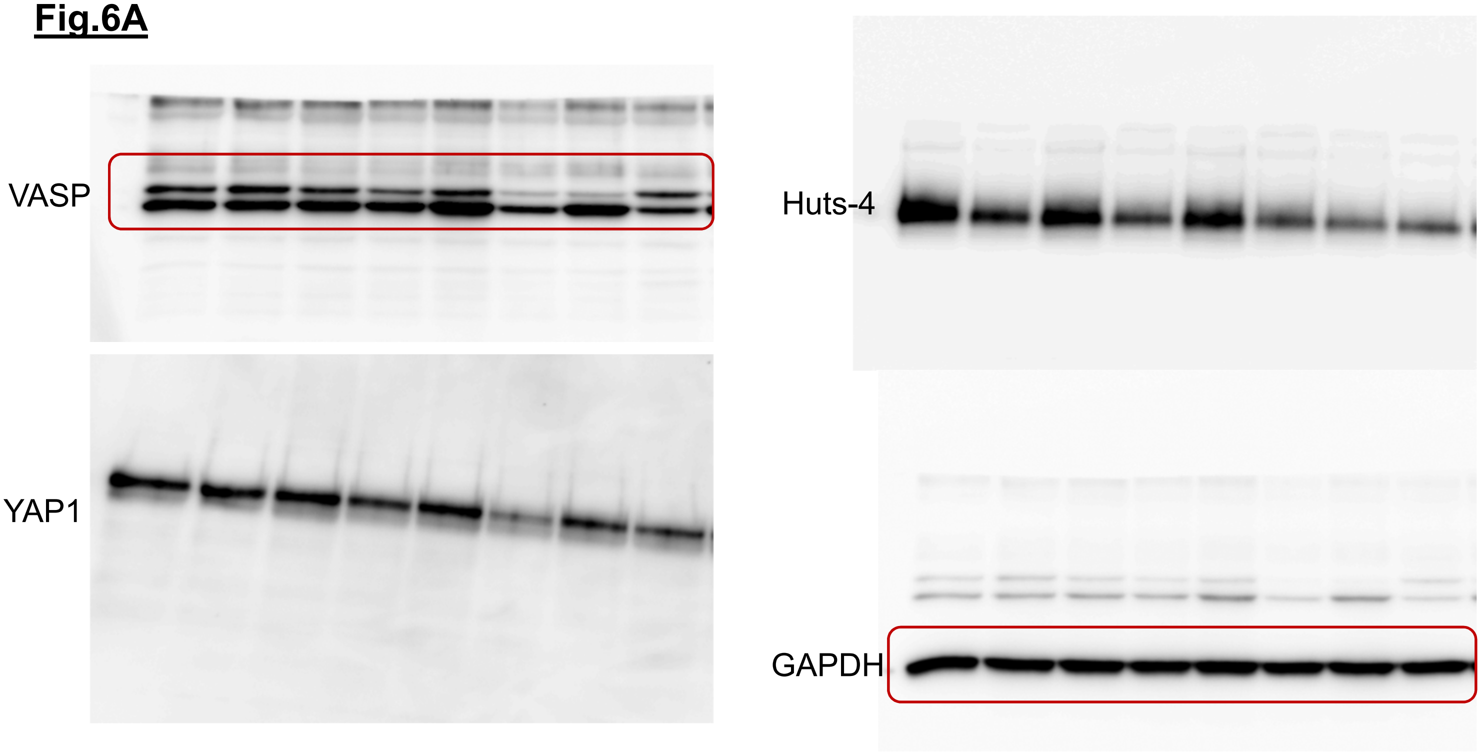


**Suppl. Fig. 2B**

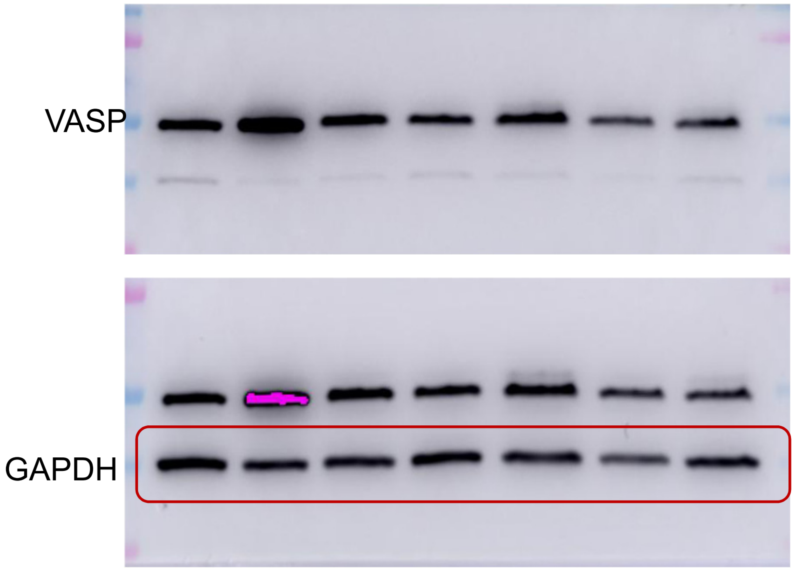

**Suppl. Fig. 3B**

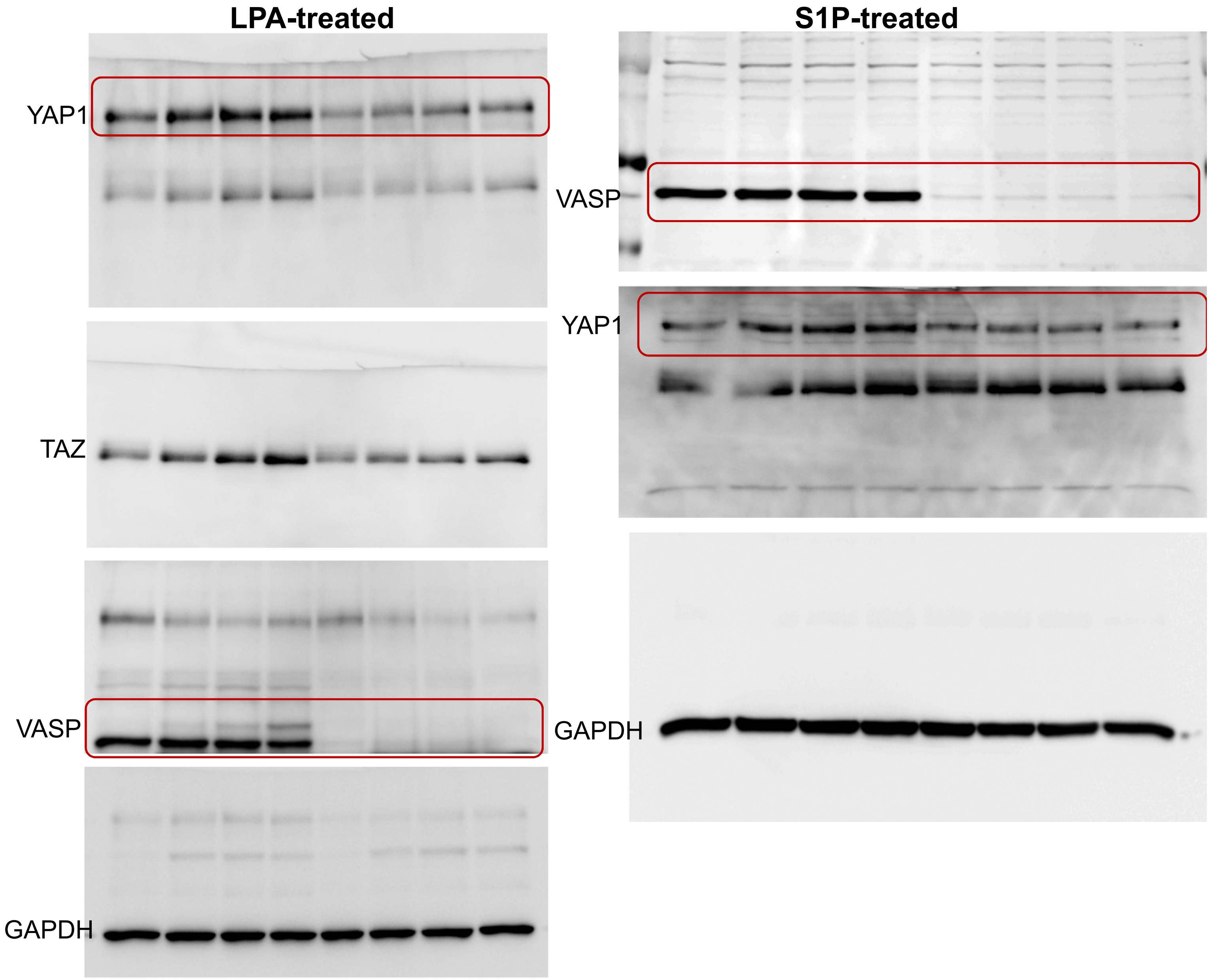

Supplement: Supplementary file 3 — Western full blot [file 41698_2017_45_MOESM3_ESM.pdf]
